# Integrated nuclear proteomics and transcriptomics identifies S100A4 as a therapeutic target in acute myeloid leukemia

**DOI:** 10.1038/s41375-019-0596-4

**Published:** 2019-10-14

**Authors:** Bader Alanazi, Chinmay R. Munje, Namrata Rastogi, Andrew J. K. Williamson, Samuel Taylor, Paul S. Hole, Marie Hodges, Michelle Doyle, Sarah Baker, Amanda F. Gilkes, Steven Knapper, Andrew Pierce, Anthony D. Whetton, Richard L. Darley, Alex Tonks

**Affiliations:** 1 Department of Haematology, Division of Cancer & Genetics, School of Medicine, Cardiff University, Cardiff, CF14 4XN Wales UK; 2 Paul O’Gorman Leukaemia Research Centre, University of Glasgow, Glasgow, G12 0ZD UK; 3 Stoller Biomarker Discovery Centre, The University of Manchester, Manchester, M20 3LJ UK; 4 Cardiff Experimental and Cancer Medicine Centre (ECMC), School of Medicine, Cardiff University, Cardiff, CF14 4XN Wales UK

**Keywords:** Apoptosis, Acute myeloid leukaemia, Preclinical research, Acute myeloid leukaemia

## Abstract

Inappropriate localization of proteins can interfere with normal cellular function and drive tumor development. To understand how this contributes to the development of acute myeloid leukemia (AML), we compared the nuclear proteome and transcriptome of AML blasts with normal human CD34^+^ cells. Analysis of the proteome identified networks and processes that significantly affected transcription regulation including misexpression of 11 transcription factors with seven proteins not previously implicated in AML. Transcriptome analysis identified changes in 40 transcription factors but none of these were predictive of changes at the protein level. The highest differentially expressed protein in AML nuclei compared with normal CD34^+^ nuclei (not previously implicated in AML) was S100A4. In an extended cohort, we found that over-expression of nuclear S100A4 was highly prevalent in AML (83%; 20/24 AML patients). Knock down of S100A4 in AML cell lines strongly impacted their survival whilst normal hemopoietic stem progenitor cells were unaffected. These data are the first analysis of the nuclear proteome in AML and have identified changes in transcription factor expression or regulation of transcription that would not have been seen at the mRNA level. These data also suggest that S100A4 is essential for AML survival and could be a therapeutic target in AML.

## Introduction

Acute myeloid leukemia (AML) is a disorder arising from developmental arrest of cells of the myeloid lineage [1]. The realization that treatment with conventional cytotoxic agents has likely reached its limits in terms of delivering patient benefit, has fueled a drive towards understanding the complex and highly heterogeneous molecular mechanisms underlying AML with the aim of delivering more targeted therapeutic approaches. Whilst common mutations have been characterized in AML [2]; it is generally acknowledged that these are unable to fully account for the highly heterogeneous nature of this disease [3]. Messenger RNA abundance can be used as an alternative strategy for target identification but mRNA levels are not powerful predictors of protein expression [4]. Further, aberrant localization of proteins to the nucleus can alter their function to induce cancer development, block in hemopoietic development, or diminish tumor suppressor function [5]. Therefore, transcriptional profiling alone is an inefficient tool for target discovery and is often combined with alternative technologies [6]. Advances in mass-spectrometry (MS) based technologies have allowed researchers to characterize and identify proteins in complex biological samples providing direct data on relative protein abundance [7].

Given that inappropriate localization of cancer-related proteins, including oncoproteins and tumor suppressor proteins may interfere with normal cellular function, we hypothesized that the developmental arrest, sustained proliferation, and prosurvival characteristics of AML blasts may in part be mediated through misexpression or mislocalization of proteins to the nucleus. The aim of this study was to identify new therapeutic targets in AML through examination of the abundance of proteins in the nuclei of AML blasts using MS proteomics. We used isobaric tags for relative and absolute quantification (iTRAQ) together with liquid chromatography-tandem MS (LC-MS/MS) to analyze the nuclear proteome of the minimally differentiated AML blasts in comparison with developmentally-matched human CD34^+^ hemopoietic stem/progenitor cells. A parallel transcriptome analysis was performed to correlate the protein data with transcriptional changes. Using this approach, we identified over 110 commonly misexpressed nuclear proteins including known abnormalities (such as WT1 and CEBPA) and novel abnormalities such as NFIC, hnHRPs. The most strongly over-expressed (novel) protein in the nucleus of AML patients was S100A4 (aka metastasin, MTS-1).

S100A4 is a ~11 kDa protein which belongs to the *S100* multigene family of calcium-binding proteins of the EF-hand type (reviewed in [8]). They have diverse roles in a variety of cellular processes including regulation of proliferation, cell cycle progression, apoptosis, differentiation, Ca^2+^ homeostasis, migration, adhesion, and transcription but little is known of its role or subcellular expression in hemopoiesis [9, 10]. S100A4 has been previously associated with poor prognosis in several solid tumors [11–14] and in leukemia [15, 16]. The functional implication of altered S100A4 expression, subcellular localization, and mechanisms of action in cancers (especially leukemia) remain unidentified. Here we identified a potential role for S100A4 and provide evidence supporting its clinical significance in AML.

## Materials and methods

### Primary cell material and cell culture

Diagnostic bone marrow or peripheral blood from AML patients and cord blood were collected with informed consent; patient clinical characteristics were outlined in Supplementary Methods. Normal human CD34^+^ cells were isolated as previously described [17].

Cell lines were obtained from ECACC^TM^ (Salisbury, UK) or ATCC (Middlesex, UK) and cultured under recommended conditions. The genetic identity of the cell lines was confirmed by short tandem repeat (STR). Cells at passages greater than twenty were not used in the experiments performed in this study. Monitoring for Mycoplasma contamination was performed using the MycoAlert Detection Kit (Sigma). S100A4 harboring a nuclear localization sequence (NLS) was expressed utilizing retroviral and lentiviral vectors co-expressing GFP as a selectable marker (Supplementary Methods). For knock down studies, Mission® shRNA vectors based on TRC(1)2-pLKO.5-puro (S100A4 shRNA and nonmammalian shRNA control) were purchased from Sigma-Aldrich, Dorset, U.K. CD34^+^ cells and cell lines were transduced and cultured as previously described [17, 18].

### Protein extraction, western blotting, and mass spectrometry

Nuclear and cytoplasmic proteins were isolated from >5 × 10^6^ fresh/frozen CD34^+^ cells and AML blasts using the Nuclear/Cytosol Fractionation Kit (Cambridge Bioscience, U.K.) following manufacturer’s instructions. A fraction of AML cells were also lysed in Trizol® for comparative mRNA analysis (Supplementary Methods) [18].

Western blotting was carried out as previously described [19] with the following antibodies: anti-S100A4 (clone D9F9D, Cell Signaling Technologies (CST), Netherlands), Histone H1 (clone AE-4, AbD Serotec, U.K.), H3 (CST), and glyceraldehyde-3-phosphate dehydrogenase (GAPDH) (6C5, Santa Cruz Biotechnology, Heidelberg, Germany).

Detailed MS proteomic methods and data analysis are shown in Supplementary Methods. The MS proteomics data have been deposited to the ProteomeXchange Consortium (http://proteomecentral.proteomexchange.org) via the PRIDE partner repository with the data set identifier PXD002799.

### GeneChip® expression profiling (GEP)

RNA isolation and GEP using Affymetrix Human Transcriptome Array 2.0 GeneChips® was performed as detailed in Supplementary Methods. All data were analyzed using Partek Genomics Suite Software using *Gene Expression* workflow (v6.6; Partek Inc., MO, USA). Significant differences were determined by ANOVA and a >±1.5 fold changes in AML vs. CD34^+^. Data is available as supplementary material at https://www.ebi.ac.uk/arrayexpress/ under the following Accession Number: E-MTAB-3873.

### Cell proliferation and viability assays

Cells were seeded in triplicate in a 96-well flat-bottom tissue-culture plate in serum-replete growth media at 1.6–2 × 10^5^ cells/mL and incubated for up to 48 h post infection. Cultures were harvested and viable (propidium iodide (PI)-negative) cells were counted by flow cytometry. For apoptosis assays, Annexin V-APC in combination with PI staining was performed.

### Flow cytometry

Flow cytometric data were acquired using an Accuri^TM^ C6 cytometer (B.D. UK). Data was analyzed using FCS Express® v6 (De Novo Software, CA). The threshold for GFP positivity was determined from the autofluorescence of GFP negative or mock transduced cells. Supplier and isotype matched control stained cells were used to determine background of labeled cells. Debris and ejected nuclei were excluded from the analysis of >10,000 events.

### Statistical analysis

Statistical significance of nonparametric data was analyzed by Mann–Whitney *U*-Test. Data represent mean ±1 SD. Calculations were performed using Minitab® v16 (Minitab Inc. USA). Network and Pathway data analysis was performed using Key Pathway Advisor and Metacore^TM^ (Clarivate Analytics, UK).

## Results

### Nuclear proteomics reveals novel proteins mis-expressed in AML

**Fig. 1:**
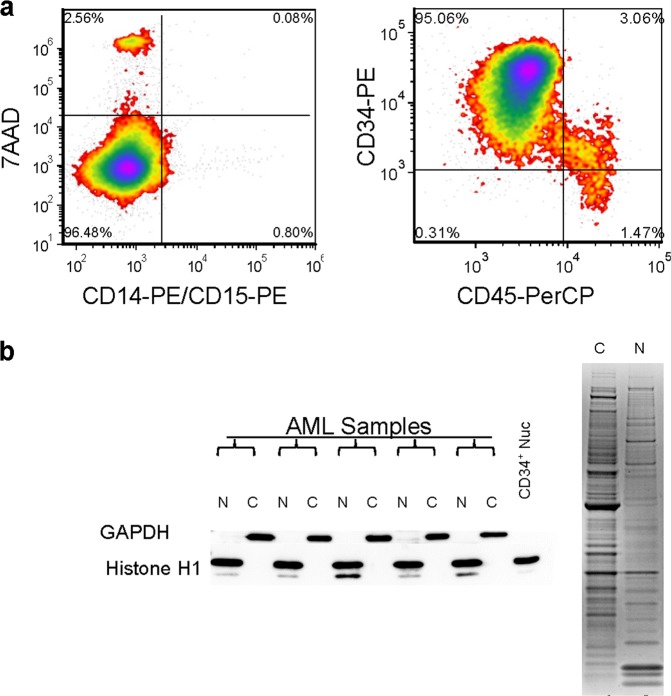
Characterization and quality control of human CD34^+^ cells and patient AML blast samples. **a** Example bivariate flow cytometric plot showing viability and immunophenotype of AML blasts used in the study (left). FAB subtype (established by morphology) was confirmed by absence of CD14/CD15 expression [20]. Data exemplifying the purity of CD34^+^ cells is shown in the right panel. Quadrants delimit background isotype staining. **b** Example chromatograms of micro-capillary electrophoresis using Agilent 2100 Bioanalyzer from representative RNA samples of AML patients. **c** Examples of fractionated protein purity and quality. Left panel shows nitrocellulose immunoblots of samples fractioned for nuclear (N), or cytoplasmic (C) proteins. Purity of the fractioned samples was assessed by immunoblotting for GAPDH and histone protein expression. Right panel shows overall protein profile and integrity quality determined through Coomassie Brilliant Blue G staining of polyacrylamide gels

We randomly selected 15 AML diagnostic samples from minimally differentiated leukemia patients (FAB type M1), to minimize variability arising from developmental differences. AML blasts were >80% viable and did not express CD14 and CD15 (Fig. 1a) (as previously described [20]). For controls we used normal human CD34^+^ cells; immunophenotypic analysis of these cells established all samples were >95% viable and 95% CD34^+^ (Fig. 1a). For transcriptome analysis, defined high quality RNA was isolated from all samples (Fig. S1b). Isolation of nuclear proteins was carried out in parallel and extract purity confirmed using western blotting of cytoplasmic and nuclear protein markers (GAPDH and histone respectively). This analysis showed little or no apparent cytoplasmic contamination in the nuclear fraction (Fig. 1b). The quality of protein extracts was confirmed by polyacrylamide gel electrophoresis which showed the absence of detectable degradation (exemplified in Fig. 1b).

To identify differentially expressed proteins, eight-channel isobaric tagging coupled with LC-MS/MS was employed to simultaneously compare nuclear proteins from AML blast cells vs. normal CD34^+^ controls (Fig. S1a for workflow). Three separate iTRAQ datasets were acquired to analyze 15 AML samples and five controls. Protein Pilot was used to normalize each ion/peptide detected within the AML sample to the control(s) within each run to provide a ratio of AML/CD34^+^ control. As expected, distributions of detectable peptide ratios from AML blasts vs. CD34^+^ (Fig. S1c) were similar, suggesting overall similarity within the proteome. A total of 4666 quantifiable proteins were identified from the nuclear samples (Table S1). Using peptides from CD34^+^ as internal controls we calculated the intra-experiment 90% confidence limits allowing us to determine if the protein(s) was significantly altered in AML blasts. We then identified frequently dysregulated proteins that changed in at least five AML patients (±>2 fold) from at least two independent MS runs. Where an AML patient sample was compared with two CD34^+^ controls we accepted a protein as ‘changed in AML’ if changes against both controls were coincident. This yielded 113 proteins of which 84 (75%) were designated nuclear proteins (Table S2).

**Fig. 2:**
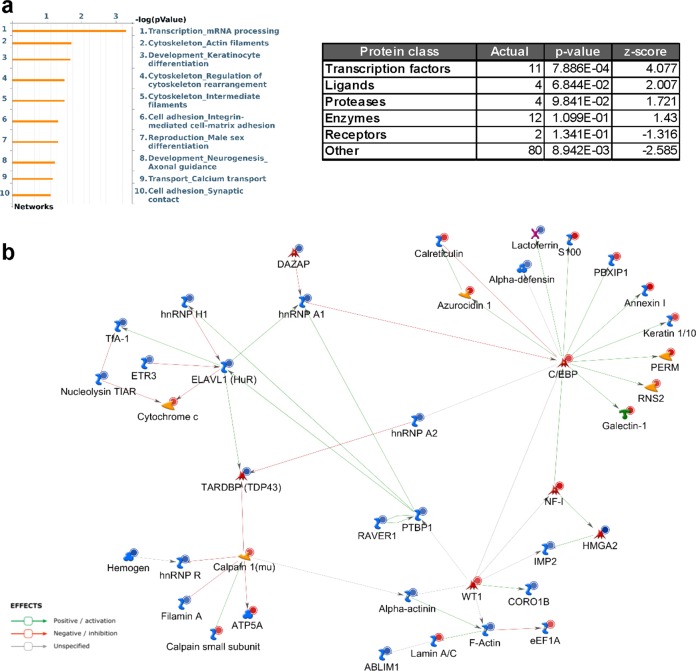
Functional enrichment analysis of protein changes observed in AML vs. normal hemopoietic CD34^+^ cells using Metacore^TM^. **a** Enrichment analyses using Process Networks (*left panel*) and enrichment by protein function (*right panel*) shows that ‘Transcription’ is the most significant Network. The Network establishes relationships between the genes from the dataset but does not cluster them according to a specific pathway. A false discovery rate (FDR) of 0.05 was applied. **b** Protein networks associated with the proteins up- or downregulated in the nuclei of AML patient blasts. The network was generated using direct interaction algorithm of MetaCore^TM^ (Clarivate Analytics). Nodes represent proteins with lines between nodes indicating protein interactions. Only connected nodes are shown. Arrow heads indicate the direction of the interaction. Node shapes represent the functional class of the proteins as shown in the graphic key (Supplementary Fig. S2b). Red and blue circles indicate up and down regulation respectively when compared with CD34^+^ nuclei. Interactome analysis using “Transcription Factor” algorithm identified CEBPA (*p* = 5.285e−08) and WT1 (*p* = 0.002825) as the most significant connected transcription factors in our protein dataset

Functional enrichment analysis of these 113 proteins showed that the most significant Gene Ontology ‘Processes’ and ‘Networks’ changed in AML patient blasts were related to Transcription, mRNA processing, and stabilization (Fig. 2a and Table S3). We observed changes in 13 heterogeneous nuclear ribonucleoproteins (hnRNP) affecting mRNA processing including: A0, A1, A2B1, A3, AB, C, D, DL, F, H1, M, R, and UL2. Enrichment by protein function showed that TF were the class of proteins most significantly enriched in our data set (Fig. 2a). We further analyzed the interactions of these 113 proteins with each other using MetaCore^TM^ analysis tool and the Direct Interaction algorithm and found a network of 40 protein interactions (Fig. 2b).

We identified three upregulated TF in leukemic cells compared with normal CD34^+^ cells (Table 1); of these, two had been previously associated with AML: CEBPA [21, 22] and WT1 [23, 24]. The third, Nuclear Factor IC (NFIC), has not been previously reported to be upregulated in AML. NFIC belongs to the NFI family which is composed of four members which differ in their ability to either activate or repress transcription (reviewed in [25]). We also identified eight down-regulated TF, of which two have been previously associated with AML: HMGA2 [26] and BCL11A [27]. The remaining six downregulated proteins are novel abnormalities in AML: DAZAP1, ILF2, ILF3, hnRPDL, MYEF2, and TARDBP (Table 1).

**Table 1:** Transcription factors frequently changed in the nucleus of patients with AML FAB M1

Gene symbol	Gene name	Frequency	Protein fold change (AML vs. CD34^+^)^a^	Normalized gene expression (fold change AML vs. CD34^+^)
NFIC	Nuclear Factor I C	6	5.5	1.5^b^
WT1	Wilms Tumor 1	8	2.9	5.6^b^
CEBPA	CCAAT/enhancer binding protein (C/EBP), alpha	7	2.9	1.7^b^
ILF3	Interleukin enhancer binding factor 3, 90 kDa	8	−2.4	−1.2
ILF2	Interleukin enhancer binding factor 2	7	−2.7	−1.2
hnRNPDL	Heterogeneous nuclear ribonucleoprotein D-like	11	−3.3	−1.0
BCL11A	B-cell CLL/lymphoma 11A (zinc finger protein)	8	−3.3	−1.4^b^
DAZAP1	TAR DNA-binding protein 43	10	−3.4	−1.2
TARDBP	DAZ associated protein 1	9	−3.8	1.3^b^
MYEF2	Myelin expression factor 2	10	−5.4	−3.7^b^
HMGA2	High mobility group AT-hook 2	14	−5.9	−3.0^b^

Proteins were selected based on those that significantly changed co-directionally ± >2 fold between normal CD34^+^ control and AML in at least 5 of the 15 patients (frequency)

^a^Calculation based solely on patients where a significant change was observed to derive the average fold change of AML vs. CD34^+^ normal control. Also shown are the fold changes of normalized gene expression data of corresponding mRNA. Positive values are upregulated in AML vs. control. Negative values downregulated in AML vs. control

^b^Genes considered to have a change in mRNA transcript abundance when analyzed by ANOVA (*P* < 0.05) and ≥ ± 1.3 fold compared with control

**Fig. 3:**
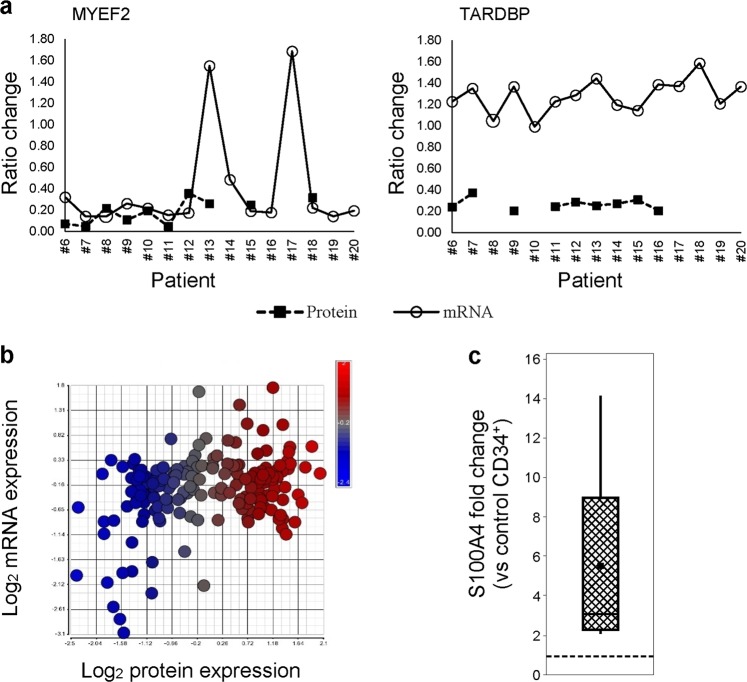
Correlation of protein and mRNA transcript expression of nuclear proteins changed in AML. **a** Protein and mRNA expression changes in two transcription factors identified to be differentially expressed between AML blasts and normal human CD34^+^ cells using LC/LC-MS/MS. Values below one are repressed in AML blasts. Some patients do not have a significant detection of protein when analyzed by LC/LC-MS/MS. **b** Correlation of nuclear protein expression with mRNA expression in nuclear proteins identified to be significantly changed between AML and CD34^+^ cells. Legend depicts level of change in protein expression in AML vs. normal CD34^+^ cells. Negative values depict lower levels of expression in AML vs. CD34^+^ cells. **c** Box and whisker plot show relative MS quantitation of S100A4 protein in expression in nuclear AML blasts vs. normal controls (*n* = 11). The dashed line represents no change to control (CD34^+^). Solid line indicates median and filled square indicates mean

To establish whether the protein expression changes identified above (Table S2) were transcriptionally driven, relative mRNA transcript abundance of AML blasts vs. CD34^+^ was analyzed. It has been previously established that mRNA expression is only predictive of protein expression in as little as 40% of genes [4]. Our data were in accord with this, with 60% agreement overall and 55% concordance in our differentially expressed TFs with a protein/mRNA fold change ≥±1.3 (Table 1 and Fig. 3a). This was not only a characteristic of TFs since ~60% of the significantly expressed proteins identified from our dataset of 113 proteins did not correlate with changes in mRNA expression (Fig. 3b). While transcription can provide at least a partial explanation for changes in protein expression, these data suggest that posttranscriptional events are of equivalent importance in regulating protein abundance in AML.

### Identification of aberrantly expressed proteins driving transcriptional change in AML

In order to functionally validate changes identified in Table 1, we carried out an unsupervised analysis of AML specific changes in the transcriptome using ANOVA and threshold analysis and coupled this with Metacore’s^TM^ network building algorithm on TF. This analysis identifies over-connected networks of known interacting proteins within the significantly changing mRNA dataset. This approach revealed 311 TF (Table S4) and of those TF, 40 were found to be significantly changed in AML. Only the changes in CEBPA and WT1 expression functionally validated the change observed at the protein level. This suggests that the abundance of these two TF proteins correlates with changes in expression of known CEBPA and WT1 target genes. This analysis is however dependent on the level of annotation in the Clarivate knowledgebase and will be subject to bias towards frequently studied proteins [28]. In light of this we tried an alternative approach to identify aberrant driver TF activity by using a causal reasoning algorithm (Key Pathway Advisor) to identify upstream regulators that are responsible for influencing the changes in nuclear protein expression observed in AML (Table S5); however, these key hubs (the most significant of which was RNF4; Fig. S2) were also not predictive of the observed protein changes.

In summary, nuclear proteomics identified transcription as being the most dysregulated process in AML with both known and novel TF being implicated (see “Discussion”). Whilst we validated the role of the former (WT1, CEBPA) by analysis of the corresponding transcriptome, we were unable to achieve this with novel TF abnormalities (probably due to relatively poor database annotation for these proteins).

### S100A4 is over-expressed in the nucleus of AML blasts

**Fig. 4:**
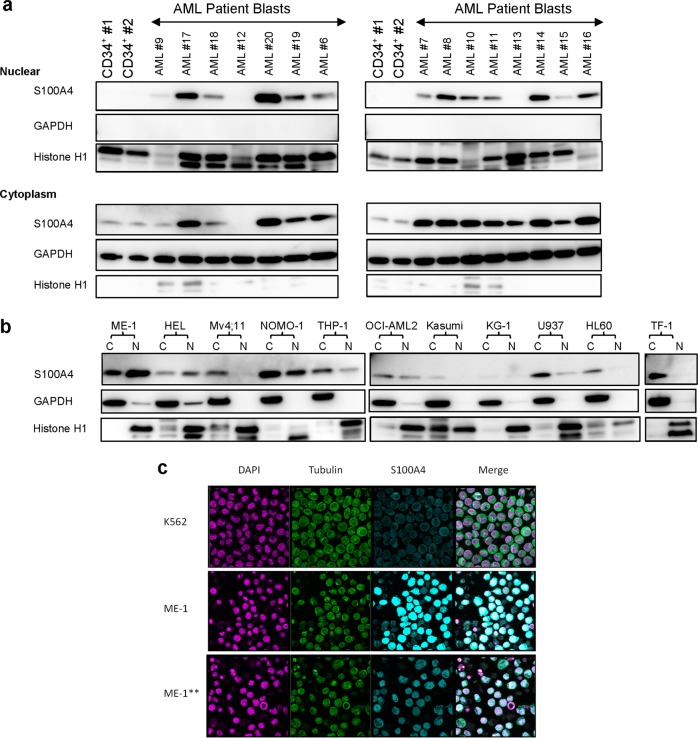
S100A4 is over-expressed in the nucleus of AML blasts. **a** Example immunoblots showing validation of S100A4 protein expression and subcellular localization in same FAB M1 patient samples analyzed by MS. Supplementary Fig. S3 shows relative S100A4 expression in cytosol and nuclear fractions. S100A4 was upregulated in the nuclei and cytoplasm of 13/15 AML patients determined by western blot. AML samples 9, 11, and 12 were derived from patient bone marrow; all others AML samples were derived from peripheral blood. **b** Expression and subcellular localization of S100A4 in a cohort of leukemia cell lines. Cytosolic (C) and nuclear (N) fractions were analyzed by GAPDH and Histone H1 to indicate the purity/relative loading of each fraction. **c** Validation and expression of endogenous S100A4 expression in K562 and ME-1 leukemic cells lines using confocal laser scanning microscopy. These cell lines have either low cytoplasmic or high nuclear protein expression of S100A4 respectively. Cells were stained with DAPI and Tubulin to define cytoplasm and nuclear compartments. Fluorescence gains were equivalent (and based on isotype controls for each panel); except for ME−1** whose gain was reduced to allow the visualization of S100A4 protein expression without saturation as shown in the middle panel

Having analyzed our data with respect to the most dysregulated process, we next investigated the most strongly changed proteins in AML compared with CD34^+^ cells (Table 2). Amongst the Top 10 changing proteins, we identified several proteins that have previously been implicated as an abnormality in AML (Supplementary Table S6). Among the previously non-implicated proteins, the highest differential expression was seen in S100A4 (Fig. 3c). S100A4 [29] was significantly upregulated in the nuclei of 11/15 AML patients with an average fold increase of 5.5 when compared with controls. We validated the expression of S100A4 in cytosolic and nuclear protein fractions using western blot from the same samples used for MS. As shown in Fig. 4a (and Fig. S3), S100A4 protein expression was observed in the nucleus of AML blasts (86%; 13/15) supporting our MS data. In contrast, nuclear expression of S100A4 was undetectable in CD34^+^ controls. Interestingly, S100A4 expression was also increased in the cytoplasm of AML blasts versus normal controls. We confirmed this data in a second cohort of patient AML blasts which also showed nuclear overexpression in seven of seven patients; and in the cytosol of nine of nine patients (Fig. S3). To establish whether S100A4 was overexpressed in a broader cohort of AML patients with blast differentiation, we immunoblotted for S100A4 in FAB-M4 subtypes (Fig. S4). Again, we found this protein to be over-expressed in the nuclei of AML blasts (4/6 patients when compared with normal human differentiated monocytes (which had undetectable levels of nuclear S100A4 expression). We also observed S100A4 expression in all leukemia cell lines analyzed with six of the ten lines having prominent nuclear expression of the protein (Fig. 4b, c).

**Table 2:** Significantly changing nuclear proteins in AML patient blasts

Gene symbol	Gene name	Frequency	Fold change (AML vs. CD34^+^)^a^	Frequency × fold change^≠^	Abnormally expressed in AML^b^
HMGA2	High mobility group protein HMGI	14	−6.0	84.7	Yes
ANXA1	Annexin A1	11	6.3	69.6	Yes
PTRF	Polymerase I and transcript release factor	11	−5.7	63.4	Yes
S100A4	Protein S100-A4	11	5.5	60.5	Not reported
LSP1	Lymphocyte-specific protein 1	13	−4.2	54.8	Yes
MYEF2	Myelin expression factor 2	10	−5.4	54.1	Yes
MPO	Myeloperoxidase	9	5.3	48.4	Yes
ANXA4	Annexin A4	11	4.20	46.2	Yes
S100A6	Protein S100-A6	11	3.89	42.8	Not reported
FLNB	Filamin B	13	−3.3	42.7	Yes

The top 10 most significant protein changes are shown based on the ^≠^product of frequency of observation and magnitude of change (independent of direction of change). At a minimum, proteins must have significantly changed ± > 2 fold between normal CD34^+^ control and AML in at least 5 of the 15 patients (Frequency) derived from Supplementary Table S2

^a^Calculation based solely on patients where a significant change was observed to derive the average fold change of AML vs. CD34^+^ normal control. Positive fold change values are upregulated in AML vs. control. Negative values downregulated in AML vs. control

^b^Literature references for proteins reported as an abnormality in AML are provided in Supplementary Table S6

**Fig. 5:**
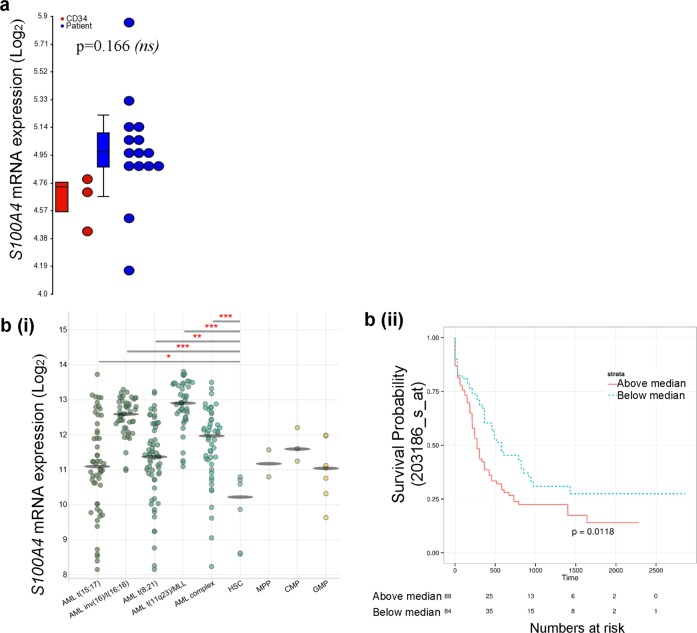
Over-expression of *S100A4* mRNA in AML. **a** Microarray data demonstrating the normalized intensity of *S100A4* mRNA (log_2_) expression in normal human CD34^+^ cells (red; *n* = 3) and FAB-M1 AML (*n* = 15). RNA was isolated from samples that underwent mass spectrometry. The transcriptome of these samples were analyzed by Affymetrix Gene expression Profiling and data analyzed using Partek Genomics Suite v6. The Pearson correlation of *S100A4* mRNA with protein expression was *r* = 0.45 (CI −0.206, 0.827). **b** *S100A4* mRNA expression data from Bloodspot [69]. (b(i)) mRNA expression level of *S100A4* in different AML subtypes vs. normal human hematopoietic developmental subsets. Human normal hematopoiesis data derived from GSE42519 [30] and human AML data derived from GSE13159 [70]. HSC, Hematopoietic stem cell Lin^−^ CD34^+^ CD38^−^ CD90^+^ CD45RA^−^; MPP, Multipotential progenitors Lin^−^ CD34^+^ CD38^−^ CD90^−^ 45RA^−^; CMP, Common myeloid progenitor cell Lin^−^ CD34^+^ CD38^+^ CD45RA^-^ CD123^+^; GMP, Granulocyte monocyte progenitors Lin^−^ CD34^+^ CD38^+^ CD45RA^+^ CD123^+^. (b(ii)) Overall survival of AML patients stratified according to *S100A4* expression level using the AML TCGA dataset [31]. Statistical significance is denoted by *P < 0.05; **P < 0.01 and ***P < 0.001 analyzed by *t*-test. ns; not significant

Upregulation of S100A4 expression in patients is also supported by our transcriptome analysis of these samples (Fig. 5) indicating that overexpression arises at least partly at a transcriptional level. Analysis of several independent datasets supports the overexpression of *S100A4* mRNA in AML (Figs. 5b(i) and S5). Further, data derived from (TCGA) [30, 31] suggests that overexpression may confer a poor prognosis (*P* = 0.0118; Fig. 5b).

In summary, nuclear overexpression of S100A4 is a very common abnormality in AML patients and AML cell lines.

### S100A4 expression is required for the growth and survival of AML cells but not for normal myeloid survival development

The above data shows S100A4 is over-expressed in the nucleus in AML. To determine whether ectopic expression of nuclear S100A4 can affect the growth and survival of CD34^+^ cells, we attempted to overexpress nuclear-targeted S100A4 in normal human hemopoietic cells (Fig. S6a). Whilst, these vectors were able to express S100A4 in HEK293T (Fig. S6b); overexpression of S100A4 could not be demonstrated in transduced CD34^+^ cells despite expressing GFP (Fig. S6c), probably due to rapid degradation of S100A4 protein in these cells.

Data from our previous microarray analysis [18] suggest differential expression of *S100A4* mRNA in normal hemopoietic cell lineages (Fig. S7). Analysis at the protein level confirmed cytosolic expression in monocytic, erythroid and (weakly) in granulocyte progenitors as well as in normal bone marrow. Nuclear S100A4 was absent in all samples (Fig. S7) suggesting that the nuclear localization of this protein in AML is aberrant. To address whether S100A4 protein was required for normal hemopoietic cell development, we knocked-down S100A4 expression in CD34^+^ cells (Fig. S8). Whilst these cells grew slightly slower (but statistically not significant), we observed no significant effect on lineage development of these cells (Fig. S8) suggesting S100A4 is not required for normal hemopoiesis. Indeed, S100A4 knock out mice do not show any obvious phenotype at birth and develop normally [32].

**Fig. 6:**
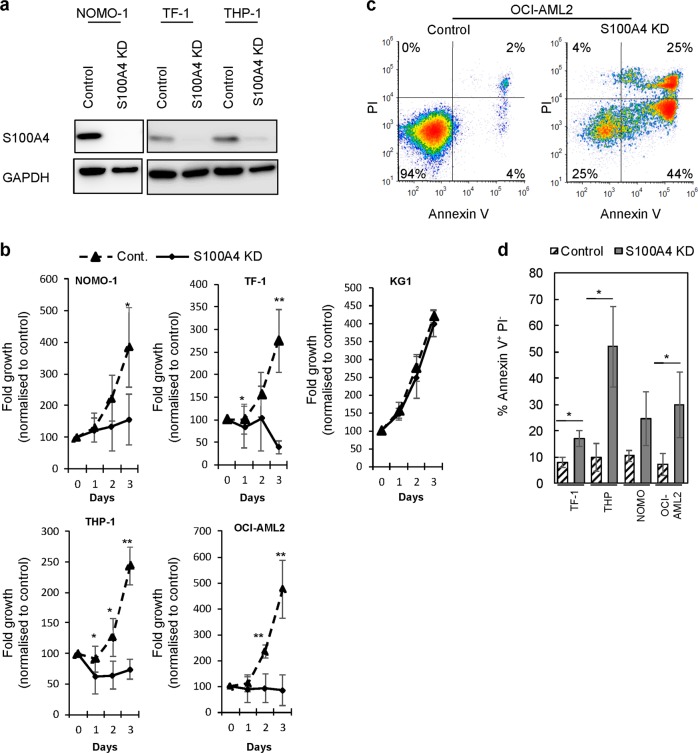
S100A4 is required for cell survival in leukemia cell lines. **a** Example western blot showing S100A4 expression in leukemia cells with S100A4 knocked down (KD; TRCN0000416498) compared with control (targeting nonmammalian gene) using shRNA. **b** Summary data showing growth of leukemia lines with S100A4 KD (TRCN0000416498) compared with control over 3 days of growth following infection (*n* = 3; except KG1 (*n* = 2). **c, d** Apoptosis was evaluated by flow cytometric analysis of APC-conjugated Annexin V binding, while simultaneously assessing membrane integrity by PI exclusion. **c** Example flow cytometric plots of S100A4 KD compared with control using OCI-AML2. Annexin V^−^ and PI^−^ negative (lower—left quadrant), annexin V^+^ and PI^−^ (lower—right quadrant) and both annexin V and PI positive (upper—right quadrant) cells were considered as the viable, early-phase apoptotic, late-phase apoptotic/necrotic cells, respectively. **d** Summary data showing the effect of S100A4 KD on Annexin V staining in leukemia cell lines following 48 h post infection. Data indicates mean ± 1 SD (*n* = 3). Statistical significance is denoted by *P < 0.05; **P < 0.001 analyzed by paired *t*-test

We next examined the consequences of knocking down S100A4 expression in leukemia cell lines (Fig. 6a). In all lines, S100A4 knockdown significantly impaired the growth of these cells (Figs. 6b and S8). Further, KD of S100A4 in AML cells (KG1) with little S100A4 expression, showed no effect on proliferation. Using flow cytometric analysis of annexin V and PI staining, the percentage of cells in early or late apoptosis was determined (Fig. 6c). In all cell lines tested, loss of S100A4 expression induced annexin V positivity (Fig. 6d) suggesting that the lack of cell growth observed above was a result of programmed cell death. Taken together these data infer that S100A4 is required for AML cell survival but not for normal cells suggesting that targeting S100A4 would be an effective strategy in this disease.

## Discussion

Given that developmental arrest is common to all AML and is mediated through misregulation of the differentiation program a number of groups have used transcriptome analyses such as oligonucleotide array or RNAseq for determining the genome wide gene expression [33–35]. However, it is becoming increasingly clear that analysis of mRNA alone is insufficient to predict biological function given that mRNA expression does not always equate to protein expression and does not identify altered subcellular localization of proteins. In this study we have therefore, carried out proteomic analysis in tandem with transcriptomics and have focused on protein changes within the nuclear compartment which are otherwise under-represented in whole cell proteomics.

Although model systems strongly implicate TF dysregulation in this disease, in patient material only a few common transcription factor abnormalities are known (e.g., PML-RARA, RUNX1-ETO, Inv16, CEPBA) and these are mainly associated with particular cytogenetic subsets of AML [36]. Therefore, large scale proteomic technologies are mainly used to quantify changes in protein abundance in in vivo or ES differentiation model systems [37–39]. However, the actual transcriptional environment in primary AML patient material characterized by the relative abundance of TF protein expression compared with normal blasts has not yet been described. However, the expression of some TF in AML and in normal CD34^+^ cells was recently described using reverse phase protein arrays using 228 validated antibodies [40]. Given that protein expression profiling patterns in AML correlate with morphologic features [41], we restricted our analysis to undifferentiated AML compared with normal human undifferentiated CD34^+^ cells to minimize changes as a result of differentiation. Using this approach, we identified significant differences in protein expression in GO processes involving mRNA stabilization. Further, we identified Networks enriched for transcription and observed significant differences in TF protein abundance in AML blasts including CEBPA and WT1. Importantly, several new and novel TF which have not been previously reported as an abnormality in AML were identified, among these were ILF2, ILF3, TARDBP, hnRPDL, DAZAP1, MYEF2, and NFIC. Interleukin enhancer binding factor (ILF) 2 encodes a 45 kDa protein and forms a complex with the 90 kDa interleukin enhancer-binding factor 3 (ILF3). This has been shown to affect the redistribution of nuclear mRNA to the cytoplasm and to negatively regulate the microRNA processing pathway [42]. TAR DNA binding protein (TARDBP) is a RNA-binding protein that has multiple functions including transcription. Little is known about TARDP but strong expression of this protein has previously been shown in the nucleolus of AML cell lines [43]. hnRNPs comprise a family of RNA-binding proteins, which are involved in processing heterogeneous nuclear RNAs into mature mRNAs and act as *trans*-factors in regulating gene expression. Within the nucleus these proteins are involved in RNA splicing, 3′-end processing, transcriptional regulation, and immunoglobulin gene recombination [44]. Recently, Gallardo et al. showed that AML patients harboring 9q deletions have decreased *HNRNPK* expression implicating the role of this protein in the development of AML [45]. NFIC belongs to NFI family of transcription factors with associated members being NFIA, NFIB, and NFIX. Regulation of cellular differentiation is reported to be the fundamental function of these members [46]. NFIC has been shown to be upregulated in several solid tumors including gastric cancer, lung squamous cell carcinoma, and colorectal cancer and is correlated with increased expression of oncogenes [47, 48].

To correlate a transcriptome signature with TF expression we analyzed the transcriptome of AML cells and used the network-building algorithm on transcription regulation from MetaCore^TM^ to examine whether the modulated genes are connected to TF. Several TF networks were identified but none of them correlated with dysregulated TF protein expression. Interestingly, 13 proteins involved in mRNA processing were shown to be dysregulated suggesting that posttranscriptional regulation of mRNAs and/or LncRNAs could play a critical role in modulating transcription. In support of this, analysis of our microarray data revealed significant differences between the transcriptome of AML cells and CD34^+^ cells particularly with the generation of mRNA transcripts through alternative splicing (data not shown).

We next examined which proteins were the most significantly changed in AML based on frequently of detection coupled with highest fold changes in our cohort of patients. We further focused on novel abnormalities by excluding proteins with known published associations with AML (Table 2). S100A4 was identified for further study, as the most significant, fold changing protein in AML blasts that is over-expressed in the nucleus of AML and has not been previously associated with AML. S100A4 belongs to the *S100* multigene family of calcium-binding proteins of the EF-hand type. These proteins are distributed into three main subgroups based on regulatory control within the extracellular or intracellular environments (or both). They have diverse roles in a variety of cellular processes including regulation of proliferation, cell cycle progression, apoptosis, differentiation, Ca^2+^ homeostasis, migration, adhesion and transcription [10, 29, 49]. S100A4 expression is found to be over-expressed in several solid tumors and has been associated with poor prognosis [11, 15, 16, 50, 51]. Interestingly, preferentially expressed antigen of melanoma (PRAME) which has previously been shown to reduce tumorigenicity of leukemic cells in vivo, has also been shown to reduce expression of S100A4 [52], particularly in those leukemias associated with favorable outcome (e.g., in leukemia’s harboring RUNX1-ETO and PML-RARα). More recently, Xu et al. demonstrated that PRAME promotes apoptotic death of leukemia cells by regulating S100A4/p53 signaling [16]. Others have previously shown S100A4 to have a key role in proliferation, cell cycle progression and cell survival in transformed cells (reviewed in [53]). S100A4 has been studied in breast cancer models which have shown that over-expression of S100A4 in nonmetastatic mammary tumor cells confers a metastatic phenotype [54]. This is consistent with earlier studies where knock down of S100A4 has been shown to reduce the self-renewal capability and tumorigenic properties of solid tumor cancer initiating cells [55, 56]. It is likely that these effects are mediated through protein binding partners of S100A4; for example, in vitro studies have shown that non-muscle myosin heavy chain IIA (NM-MHC IIA) can bind directly to S100A4 and modulate the interaction between non-muscle myosin and actin, resulting in cytoskeletal rearrangement and increased migration [57]. This would be consistent with pathway changes we observed in the nucleus of AML blasts (Fig. 2a). S100A4 has also been shown to interact directly with p53 in the nucleus and induce MDM2-dependent p53 degradation [58]. In this latter study, S100A4 knock down leads to a p53-dependent cell cycle arrest and increased cisplatin-induced apoptosis. However, in this study we did not detect p53 bound to S100A4 (data not shown). Interestingly S100A4 has previously been found to be a downstream target of CEBPA [59] and SP1 [60, 61], key hub targets identified through our transcriptome analysis.

Whilst S100A4 has been widely studied in solid tumors very little is known of its role in hematological malignancies. We show for the first time that normal CD34^+^ cells and myeloid differentiated lineages express this protein in the cytosol. Knocking down expression of S100A4 in AML lines results in cell death through induction of apoptosis and hence is an attractive target for cancer therapy particularly in AML given that normal cells would be spared [62]. Increasing evidence suggests that expression and subcellular localization of several S100 proteins is different between physiological and pathological conditions. Indeed, we also observed nuclear expression changes in S100A6 and S100A11 in our data set. Interestingly, S100A8 and S100A9 have previously been shown to be abundant in myeloid cells and associated with poor prognosis in AML [63–65]; these studies focused on total expression and not subcellular expression. S100A4 is predominantly a cytosolic protein under normal physiological conditions but few studies have identified this protein in the nucleus of transformed cells [66–68]. In our cohort of AML, there were very few cell lines and patient derived blasts with no expression of S100A4 in the nucleus. It remains to be determined whether S100A4 is mislocalized to the nucleus in AML or is a result of the high expression of this protein in AML. S100A4 has been shown to undergo several posttranslational modifications in other contexts, including oxidative modification or sumoylation, which can modulate intracellular localization [67]. Nuclear localization of S100A4 in AML would facilitate regulation of gene transcription either through direct DNA binding, or through interaction with other DNA‐binding proteins as previously described [8]. However, we found little evidence of this in our transcriptome data and Metacore^TM^ analysis; though this analysis is dependent on the level of annotation in the Clarivate knowledgebase [26]. We are currently investigating the binding partners of S100A4 that are responsible for the shuttling of this protein between the cytosplasm and nucleus or whether S100A4 binds to nuclear proteins that enhance its retention in the nucleus using proteomics.

In summary, we report the first study to use iTRAQ proteomic analysis coupled with mRNA GEP to identify several proteins that are expressed or repressed in the nucleus of AML blasts. One of these proteins, S100A4, is essential for AML cell growth and survival suggesting that therapeutically targeting S100A4 would be an effective strategy while sparing normal hemopoietic cells.

## Supplementary information

Supplemental Methods

Supplemental Figures

Table S1

Table S2

Table S3

Table S4

Table S5

Table S6
