# Supplementary material for: Integrated nuclear proteomics and transcriptomics identifies S100A4 as a therapeutic target in acute myeloid leukemia
Source: Leukemia. 2019 Oct 14;34(2):427–40. doi: 10.1038/s41375-019-0596-4 (PMC6995695; doi:10.1038/s41375-019-0596-4)
Supplement: Supplementary file 1 — Supplemental Methods [file 41375_2019_596_MOESM1_ESM.docx]

# Supplemental Methods

## Primary cell material and cell culture

Diagnostic bone marrow or peripheral blood from AML patients and normal human cord blood from healthy donors were collected with informed consent; patient clinical characteristics outlined below. For expression of S100A4 in normal human bone marrow, mononuclear cells were purchased from Stem Cell technologies.

*Sample characteristics included in LC/LC-MS/MS proteomic and microarray analyses*.

| Sample  number | Sample  type | CD34^+ (%)^ | Array  Cel file | Age  (y) | Sex | Cytogenetics | Mutations tested  (NPM1/FLT3-TKD/FLT3-ITD) | Diagnosis | WBC *(x10^9^/L)* | Treatment |
| --- | --- | --- | --- | --- | --- | --- | --- | --- | --- | --- |
| *CB #1* | CB-CD34 | >95 | n/a | n/a | n/a | n/a | n/a | n/a | n/a | n/a |
| *CB #2* | CB-CD34 | >95 | n/a | n/a | n/a | n/a | n/a | n/a | n/a | n/a |
| *CB #3* | CB-CD34 | >95 | CM1.CEL | n/a | n/a | n/a | n/a | n/a | n/a | n/a |
| *CB #4* | CB-CD34 | >95 | CM2.CEL | n/a | n/a | n/a | n/a | n/a | n/a | n/a |
| *CB #5* | CB-CD34 | >95 | CM3.CEL | n/a | n/a | n/a | n/a | n/a | n/a | n/a |
| *AML #6* | AML (PB) | 0 | CM4.CEL | 63 | F | Failed | WT / WT / WT | De Novo | 46 | Daunorubicin 35 + cytarabine 200 |
| *AML #7* | AML (PB) | 0 | CM5.CEL | 56 | M | 46,XY [20] | Type D / WT / ITD | De Novo | 64 | ADE+GO |
| *AML #8* | AML (PB) | 5 | CM6.CEL | 68 | F | 46,XX [20] | Type D / WT / ITD | De Novo | 114 | DA  (3 courses) |
| *AML #9* | AML (BM) | 0 | CM7.CEL | 77 | M | 46,XY [20] | Type A / WT / WT | De Novo | 130 | LDAC  +ATO |
| *AML #10* | AML (PB) | 0 | CM8.CEL | 49 | M | NK | Type A / WT / ITD | De Novo | 153 | DA60 + GO |
| *AML #11* | AML (BM) | 0 | CM9.CEL | 55 | F | 46,XX [20] | Type B / WT / WT | De Novo | 77 | DA60 |
| *AML #12* | AML (BM) | 19 | CM10.CEL | 37 | F | 46,XX [20] | WT / WT / WT | De Novo | 9 | ADE+GO |
| *AML #13* | AML (PB) | 12 | CM11.CEL | 17 | M | 46,XY [20] | WT / WT / WT | De Novo | 294 | FLAG-IDA+GO |
| *AML #14* | AML (PB) | 40 | CM12.CEL | 48 | F | 46,XX,t(9;11)  (p22;q23) [10] | WT / WT / WT | De Novo | 46 | ADE |
| *AML #15* | AML (PB) | 75 | CM13.CEL | 65 | M | 47,XY,+8 [5]/46,XY [17] | WT / WT / WT | De Novo | 9 | DA (3 courses) |
| *AML #16* | AML (PB) | 44 | CM14.CEL | 54 | F | 46,XX [15] | Type A / WT / ITD | De Novo | 39 | ADE+GO |
| *AML #17* | AML (PB) | 0 | CM15.CEL | 65 | M | NDS | WT/ D835Y / WT | De Novo | 42 | n/a |
| *AML #18* | AML (PB) | 0 | CM16.CEL | 65 | M | 46,XY [20] | Type A / WT / ITD | De Novo | 36 | n/a |
| *AML #19* | AML (PB) | 0 | CM17.CEL | 55 | M | 46,XY [5] | Type A / D835Y / ITD | De Novo | 254 | Daunorubicin 35 + cytarabine 200 |
| *AML #20* | AML (PB) | 0 | CM18.CEL | 59 | M | 47,XY,+Y[20] | Uknown/ WT/ WT | De Novo | 57 | ADE |

ATO, Arsenic Trioxide; CB, Cord Blood; PB, Peripheral Blood; BM, Bone Marrow; DA, Daunorubicin/Cytarabine; FLAG-Ida, Fludarabine, Cytarabine, GMCSF-Idarubicin; GO, Myelotarg; N/A, Not Applicable; NK, Normal Karyotype; WBC, White Blood Cell Count; LD, Low Dose.

AML blasts were used from cryopreserved material, upon thawing, AML blast viability and cell surface phenotype were analyzed by flow cytometry to support French-American-British (FAB) classification. We did not observe any difference in expression of S100A4 in fresh *vs* fresh/frozen AML blasts (data not shown). AML patient blast FAB-M1 subtype was confirmed using CD14-PE and CD15-PE (Biolegend, San Diego, CA). In our experimental design we restricted the analysis to minimally differentiated FAB-M1 since this subtype has little developmental heterogeneity and would also be developmentally matched to normal human CD34^+^ derived from neonatal cord blood (CB) (thereby excluding differences which might rise purely from developmental variation). Previously, we found that CB-derived quiescent CD34^+^ cells were more transcriptionally matched to AML blasts than cytokine-induced CB cells [1]. The final experimental design comprised nuclear protein and RNA extracts from AML blast samples (n=15) and CD34^+^ from healthy donors (n=5). CD34^+^ controls were used as the comparator and to normalize data from each MS run. For mRNA Affymetrix analysis, all sample extracts were analyzed as a single batch to minimize hybridization variations.

## Plasmid and shRNA sequences

In over-expression studies, S100A4 cDNA (NM_019554.2) was purchased from Eurofins Scientific (MWG; Germany) and directionally sub-cloned using *BamH1* and *EcoR1* restriction sites into retroviral and lentiviral vectors co-expressing GFP as a selectable marker (PINCO [2] and pHIV, Addgene Cambridge, Massachusetts, USA). To target expression of S100A4 to the nucleus, an additional vector was prepared where the N terminus encoded a 1x or 3x nuclear localization sequence (NLS) “GATCCAAAAAAGAAGAGAAAGGTA” [3].

For knock down studies, Mission^®^ shRNA vectors based on TRC(1)2-pLKO.5-puro (S100A4 shRNA and non-mammalian shRNA control) were purchased from Sigma-Aldrich, Poole, UK and optimised in NOMO-1 cells. The most efficient KD sequence of S100A4 (TRCN0000416498) was used in combination with the following vector pLV-EGFP:T2A:Puro-U6 purchased from VectorBuilder (California, USA).

| Vector | Target Sequence information | Source |
| --- | --- | --- |
| shRNA Control | Scramble shRNA | Mission® (Sigma, UK) ^#^ |
| shRNA S100A4 clone 1 | [TRCN0000437516](https://portals.broadinstitute.org/gpp/public/clone/details?cloneId=TRCN0000437516) | Mission® (Sigma, UK) ^#^ |
| shRNA S100A4 clone 2 | [TRCN0000438093](https://portals.broadinstitute.org/gpp/public/clone/details?cloneId=TRCN0000438093) | Mission® (Sigma, UK) ^#^ |
| shRNA S100A4 clone 3 | [TRCN0000416498](https://portals.broadinstitute.org/gpp/public/clone/details?cloneId=TRCN0000416498) | Mission® (Sigma, UK) ^#^ |
| shRNA S100A4 clone 4 | [TRCN0000446826](https://portals.broadinstitute.org/gpp/public/clone/details?cloneId=TRCN0000446826) | Mission® (Sigma, UK) ^#^ |
| shRNA S100A4 clone 5 | [TRCN0000053608](https://portals.broadinstitute.org/gpp/public/clone/details?cloneId=TRCN0000053608) | Mission® (Sigma, UK) ^#^ |
| pLV[shRNA S100A4]-EGFP:T2A:Puro-U6 | [TRCN0000416498](https://portals.broadinstitute.org/gpp/public/clone/details?cloneId=TRCN0000416498) | VectorBuilder, CA, USA |
| pLV[shRNA Control]-EGFP:T2A:Puro-U6 | Scramble shRNA | VectorBuilder, CA, USA |

^#^<http://www.sigmaaldrich.com/life-science/functional-genomics-and-rnai/shrna/library-information/vector-map.html#pLKO>

## Protein extraction and western blotting validation

The nuclear protein pellet obtained post cytosol extraction was snap frozen and thawed in three freeze-thaw cycles, following which the pellet was incubated on ice for 30-60 min in the presence of 50U Benzonase (Merck-Millipore, UK). Post incubation the pellet was lysed in triethylammonium bicarbonate (TEAB) buffer (0.5M TEAB, 0.05% SDS, Protease Inhibitor cocktail and Phosphatase inhibitor cocktail) (Sigma-Aldrich, UK) for 30 min on ice, vortexing every 10 min. The lysate was centrifuged at 10 000×g at 4^o^C for 10 min. The supernatant containing the nuclear proteins was aspirated to a fresh-chilled tube. Proteins were quantified using standard Bradford assay as described previously [4].

Confocal microscopy was carried out as previously described using Anti-S100A4 Alexa Fluor^®^ 647 in conjunction with anti-αtubulin Alex Fluor^®^594 (Abcam) to visualize cytoplasmic/nuclear S100A4 expression [5].

## iTRAQ labelling and reversed phase Chromatography

40-60 μg of protein from each sample was reduced using 0.1 v of 50 mM tris-(2-carboxyethyl) phosphine (Sigma) by incubation for 1 h at 60^o^C followed by alkylation using 0.05 v of 200 mM methylmethanethiosulphate (MMTS) in isopropanol (Life Technologies, Paisley, UK). Samples were digested with Trypsin using an enzyme to sample ratio of 1:10 (*w/w*) overnight at 37^o^C. Samples were subsequently labeled with iTRAQ reagent (Applied Biosystems, Cheshire, UK) according to the manufacturer’s instructions and pooled prior to analysis.

iTRAQ labelled samples were fractionated offline at high pH using an Agilent 1200 series (Agilent Technologies, Cheshire, UK) on a Reverse Phase (RP) chromatography column (Zorbax C18 3 µm 150/4.6). Peptides were separated by applying a solvent gradient of high pH buffer A (0.1% Ammonium hydroxide, adjusted to pH 10.5 with formic acid) and high pH buffer B (0.1% Ammonium hydroxide, 99.9% acetonitrile). Initially, the gradient was run at 700 µl/min using 99.5% high pH buffer A and 0.5% high pH buffer B. Over 30 min, high pH buffer B was increased to 50% followed by 75% for 4 min prior to reducing back down to 0.5%. Fifteen second fractions were collected for the duration of the gradient and then vacuum dried.

## Mass Spectrometry data acquisition, search parameters and acceptance criteria

Mass spectrometry was carried out using an AB Sciex 5600 Triple ToF or LTQ Orbitrap Velos for the identification of peptides. For analysis using 5600 triple ToF, dried peptide fractions were re-suspended in 15 µl of 3% (*v/v*) acetonitrile, 0.1% (*v/v*) formic acid and 20 mM citric acid. For each analysis, the peptide sample was loaded onto a nanoACQUITY UPLC Symmetry C18 Trap and flow rate was set to 15 µl/min of 3% (*v/v*) acetonitrile, 0.1% (*v/v*) formic acid and 20 mM citric acid for 5 min. Analytical separation of the peptides was performed using nanoACQUITY UPLC BEH C18 Column, 1.7 µm, 75 µm × 250 mm. The peptides were separated over a 91 min solvent gradient from 3% (*v/v*) acetonitrile, 0.1% (*v/v*) formic acid to 40% (*v/v*) acetonitrile, 0.1% (*v/v*) formic acid. Data acquisition was carried out using an information dependent acquisition (IDA) 5600 Triple ToF (Sciex, Framingham) wherein, for each cycle, the 20 most abundant multiply charged peptides (2^+^ to 4^+^) above a 150 count threshold in the MS scan with m/z between 400 and 2000 were selected for MS/MS. Each ion was dynamically excluded (±50 mmu) for 90 seconds. Data was also acquired using the LTA Orbitrap Velos wherein sample was loaded on the Acclaim PepMap µ-Precolumns (500µm x 5mm) and flow rate was set to 30 µl/min of 3% (*v/v*) acetonitrile, 0.1% (*v/v*) formic acid for 5 min. Peptides were analytically separated using Acclaim PepMap RSLC C18 Columns (2 µm, 75 µm×500 mm). Briefly, peptides were separated over a 91 min solvent gradient, the constitution of the liquid phase went from 3% (*v/v*) acetonitrile, 0.1% (*v/v*) formic acid to 40% (*v/v*) acetonitrile, 0.1% (*v/v*) formic acid on-line to the Orbitrap. Data was acquired at a resolution of 60,000 at m/z 400 with an automatic gain control (AGC) target of 10^6^ in which each full scan was followed by selecting 10 most intense ions. MS/MS analysis was performed at 7500 resolution in the Orbitrap. Selected ions were excluded from further analysis for 60 s and ions with an unassigned charge of +1 were rejected.

All MS data was submitted to ProteinPilot^TM^ software 4.1, revision number 460, Paragon^TM^ Algorithm 4.0.0.0, 459 with default settings (AB SCIEX, Framingham, USA) for iTRAQ reporter ion quantification, and database searching. The software has presets to include for the allowance of one missed cleavage, 8plex iTRAQ and MMTS modifications. The data was processed by a ‘thorough’ search against the ens_homo_sapiens_core_70 human database containing 532146 sequence entries. The mass spectrometry proteomics data have been deposited to the ProteomeXchange Consortium (http://proteomecentral.proteomexchange.org) via the PRIDE partner repository with the data set identifier PXD002799.

## RNA isolation and Affymetrix mRNA GeneChip^®^ expression profiling (GEP)

Patient AML blast and CD34^+^ cells were washed twice in ice cold PBS and high-quality total RNA was extracted by lysis in Trizol^®^ according to the PureLink^®^ RNA Mini Kit manufacturer’s instructions. RNA quality, quantity and purity were assessed using Agilent RNA 6000 Nano Kit on the Agilent 2100 Bioanalyzer (Agilent Technologies, UK) following manufacture’s instruction. Only high-quality RNA (defined as having a RNA Integrity number (RIN) >7.0 and A_260/280_ ratio of ~2.0) was used in Affymetrix GEP. cDNA was generated from 100 ng total RNA using the Ambion WT Expression Kit following manufacturers protocol (Applied Biosystem, UK). cDNA was subsequently fragmented and labelled using the Affymetrix GeneChip^®^ WT Terminal Labelling Kit (Affymetrix, UK) and hybridized to Affymetrix Human Transcriptome Array 2.0 GeneChip^®^.  GeneChips^®^ were subsequently washed and stained using the Affymetrix GeneChip^®^ Hybridization, Wash and Stain Kit (Affymetrix) followed by scanning on the GeneChip^®^ Scanner 3000 as part of the Affymetrix GeneChip^®^ profiling service at the School of Medicine, Cardiff University, UK. All Affymetrix data were analyzed using Partek Genomics Suite Software using PGS *Gene Expression* workflow (Version 6.6; Partek Inc., MO, USA). For Partek analyses CEL files were imported using Affymetrix annotation files (NetAffx, Version na31. hg19). The RMA algorithm was used with adjustments made for GC content and probe sequence on pre-background-subtracted values. We used background correction, quantile normalization, log_2_ transformation, and median polishing for summarization. All image plots passed visual inspection. Hybridization controls had signal increases following concentration. Labelling control signal strengths followed the order Lys < Phe < Thr < Dap. Signal histograms and box plots were examined for raw and processed data. Significant differences were determined by ANOVA and a >± 1.5 fold changes in AML vs CD34^+^. Affymetrix data is available as supplementary material at https://www.ebi.ac.uk/arrayexpress/ under the following Accession Number: E-MTAB-3873.

## References

(1) Munje CR, Hills RK, Whetton A, Burnett A.K, Darley RL, Tonks A. Cord blood-derived quiescent CD34^+^ cells are more transcriptionally matched to AML blasts than cytokine-induced normal human hematopoietic CD34^+^ cells. *Gene Expression* 2014 Dec 6;16:169-75.

(2) Grignani F, Kinsella T, Mencarelli A, Valtieri M, Riganelli D, Grignani F, *et al*. High-efficiency gene transfer and selection of human hematopoietic progenitor cells with a hybrid EBV/retroviral vector expressing the green fluorescence protein. *Cancer Res* 1998 Jan 1;58(1):14-9.

(3) Fischer-Fantuzzi L, Vesco C. Cell-dependent efficiency of reiterated nuclear signals in a mutant simian virus 40 oncoprotein targeted to the nucleus. *Mol Cell Biol* 1988 Dec;8(12):5495-503.

(4) Hole PS, Zabkiewicz J, Munje C, Newton Z, Pearn L, White P, *et al*. Overproduction of NOX-derived ROS in AML promotes proliferation and is associated with defective oxidative stress signaling. *Blood* 2013 Nov 7;122(19):3322-30.

(5) Morgan RG, Pearn L, Liddiard K, Pumford SL, Burnett AK, Tonks A, *et al*. gamma-Catenin is overexpressed in acute myeloid leukemia and promotes the stabilization and nuclear localization of beta-catenin. *Leukemia* 2013 Feb;27(2):336-43.
