# Supplementary material for: Integrated nuclear proteomics and transcriptomics identifies S100A4 as a therapeutic target in acute myeloid leukemia
Source: Leukemia. 2019 Oct 14;34(2):427–40. doi: 10.1038/s41375-019-0596-4 (PMC6995695; doi:10.1038/s41375-019-0596-4)
Supplement: Supplementary file 2 — Supplemental Figures [file 41375_2019_596_MOESM2_ESM.pdf]

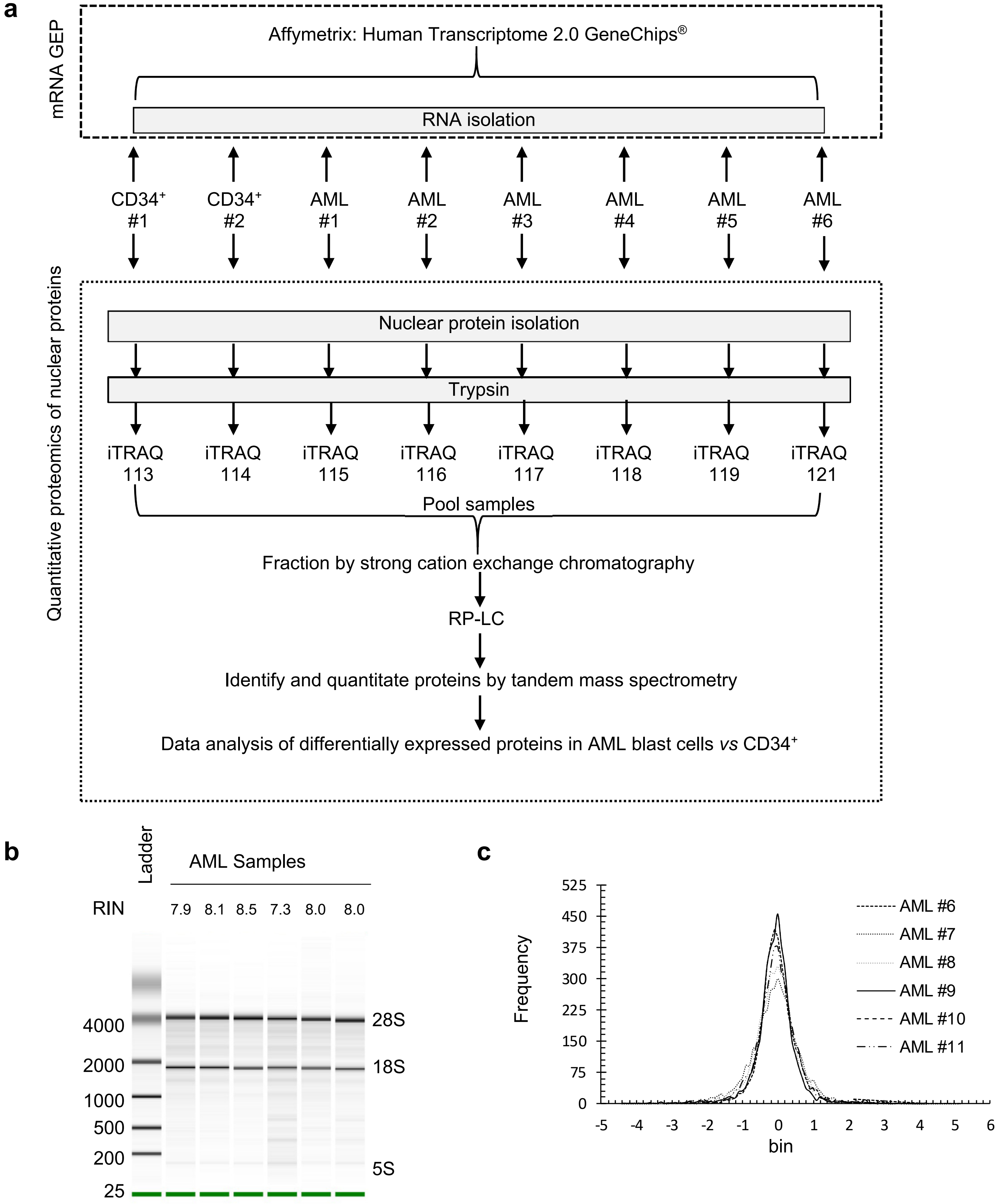

Supplemental Figure S1. Schematic of typical work flow for proteome and transcriptome analysis. **(a)** RNA was isolated from CD34⁺ cells for mRNA transcriptome analysis using Affymetrix HTA 2.0 GeneChips®. Example chromatograms of micro-capillary electrophoresis using Agilent 2100 Bioanalyzer from representative RNA samples of AML patients are shown in ***(b)***. A fraction of AML samples was also used for 8 channel isobaric tagging to allow relative quantification (iTRAQ) analysis of nuclear protein. **(c)** Three runs of LC/LC-MS/MS were performed. Representative histograms displaying the distribution of the protein ratios (AML/CD34⁺ (1)) in a typical run are shown. RIN, RNA integrity number; GEP, Gene Expression Profiling; #, sample number.

a

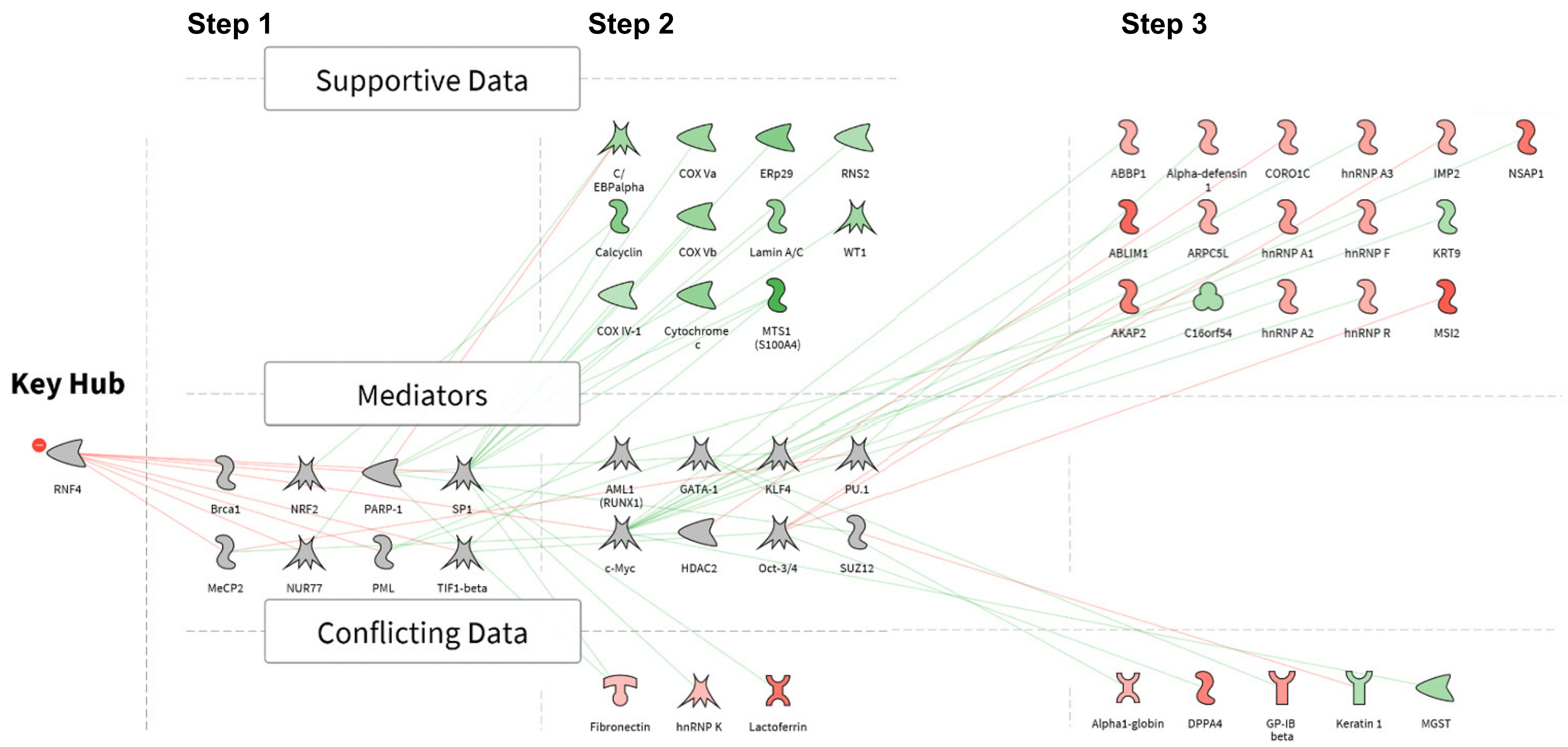

Supplemental Figure S2. Identification of RNF4 as a Key Hub. **(a)** The most significant Key Hub identified (that mediate the changes observed in our changing protein list) encodes ring finger 4 (RNF4)(P=9.391^{-E4}). Clarivate Analytics Causal Reasoning algorithm was used to identify Key hubs which predict key protein activity changes, that could be the root cause of changes observed in step 1 or via 'mediators' to affect change in steps 2 and 3. RNF4 is reasoned not to be expressed (active) denoted by 'negative'. Gray indicates protein is not in the "protein changing input list". Protein changes deemed to be supportive and align with predictive activity are listed at the top. Protein changes that conflict with predicted activity are aligned at the bottom. RNF4 is an E3 ubiquitin-protein ligase which contains a RING finger motif acting as a transcription regulator by modifying protein abundance through polyubiquitination of substrates and their subsequent targeting to the proteasome. A key TF regulated by RNF4 is SP1 which has the most connectivity to changing proteins within AML, including 9 significantly upregulated proteins which supports causal reasoning. However, we did not detect significant changes in nuclear protein expression of this TF in our MS study. **(b)** Legend overpage.

b

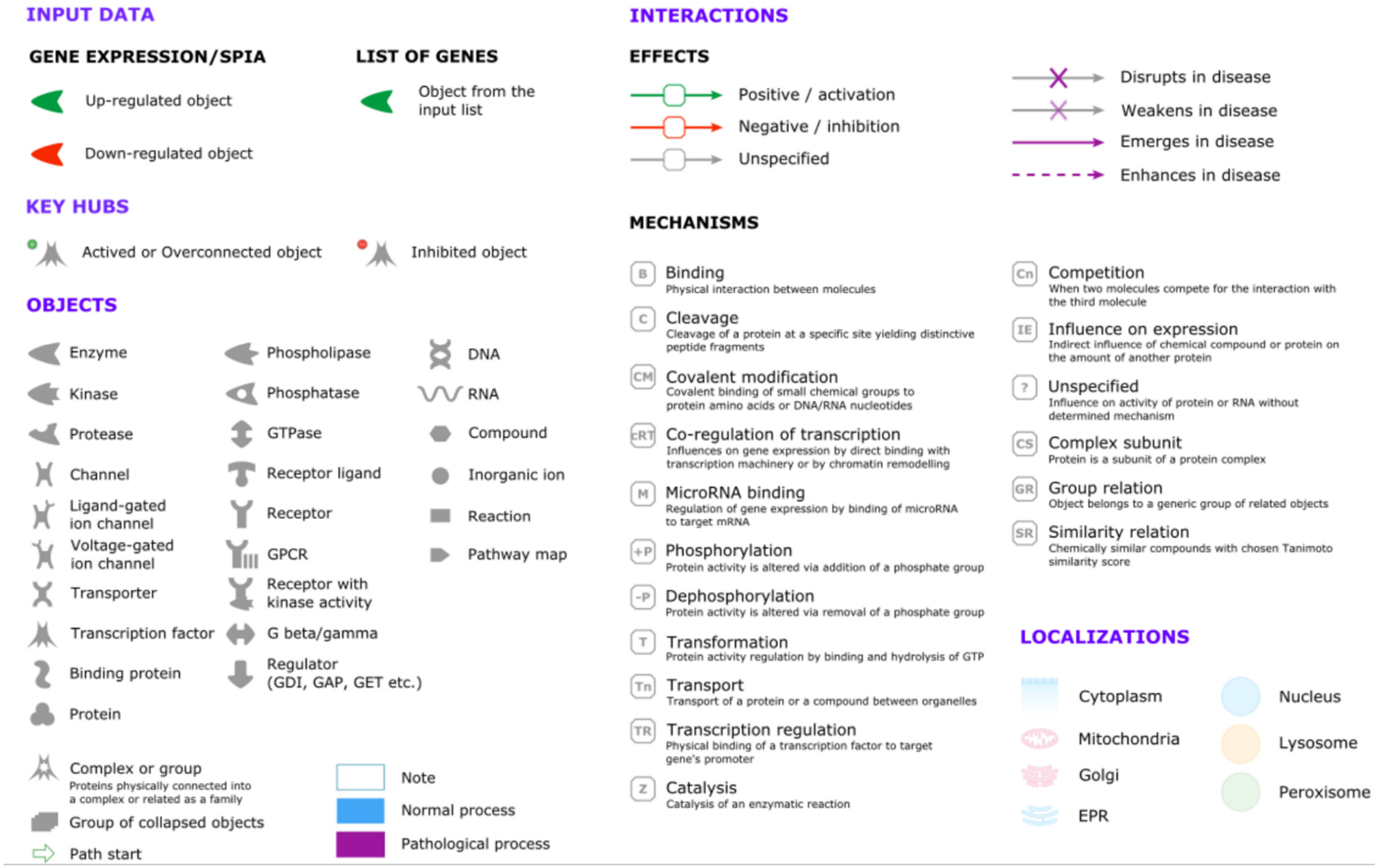

INPUT DATA
GENE EXPRESSION/SPIA
Up-regulated object
Down-regulated object
LIST OF GENES
Object from the input list
KEY HUBS
Actived or Overconnected object
Inhibited object
OBJECTS
Enzyme
Kinase
Protease
Channel
Ligand-gated ion channel
Voltage-gated ion channel
Transporter
Transcription factor
Binding protein
Protein
Phospholipase
Phosphatase
GTPase
Receptor ligand
Receptor
GPCR
Receptor with kinase activity
G beta/gamma
Regulator (GDI, GAP, GET etc.)
DNA
RNA
Compound
Inorganic ion
Reaction
Pathway map
Complex or group
Proteins physically connected into a complex or related as a family
Group of collapsed objects
Path start
Note
Normal process
Pathological process
INTERACTIONS
EFFECTS
Positive / activation
Negative / inhibition
Unspecified
Disrupts in disease
Weakens in disease
Emerges in disease
Enhances in disease
MECHANISMS
B
Binding
Physical interaction between molecules
C
Cleavage
Cleavage of a protein at a specific site yielding distinctive peptide fragments
CM
Covalent modification
Covalent binding of small chemical groups to protein amino acids or DNA/RNA nucleotides
CRT
Co-regulation of transcription
Influences on gene expression by direct binding with transcription machinery or by chromatin remodelling
M
MicroRNA binding
Regulation of gene expression by binding of microRNA to target mRNA
+P
Phosphorylation
Protein activity is altered via addition of a phosphate group
-P
Dephosphorylation
Protein activity is altered via removal of a phosphate group
T
Transformation
Protein activity regulation by binding and hydrolysis of GTP
Tn
Transport
Transport of a protein or a compound between organelles
TR
Transcription regulation
Physical binding of a transcription factor to target gene's promoter
Z
Catalysis
Catalysis of an enzymatic reaction
Cn
Competition
When two molecules compete for the interaction with the third molecule
IE
Influence on expression
Indirect influence of chemical compound or protein on the amount of another protein
?
Unspecified
Influence on activity of protein or RNA without determined mechanism
CS
Complex subunit
Protein is a subunit of a protein complex
GR
Group relation
Object belongs to a generic group of related objects
SR
Similarity relation
Chemically similar compounds with chosen Tanimoto similarity score
LOCALIZATIONS
Cytoplasm
Mitochondria
Golgi
EPR
Nucleus
Lysosome
Peroxisome

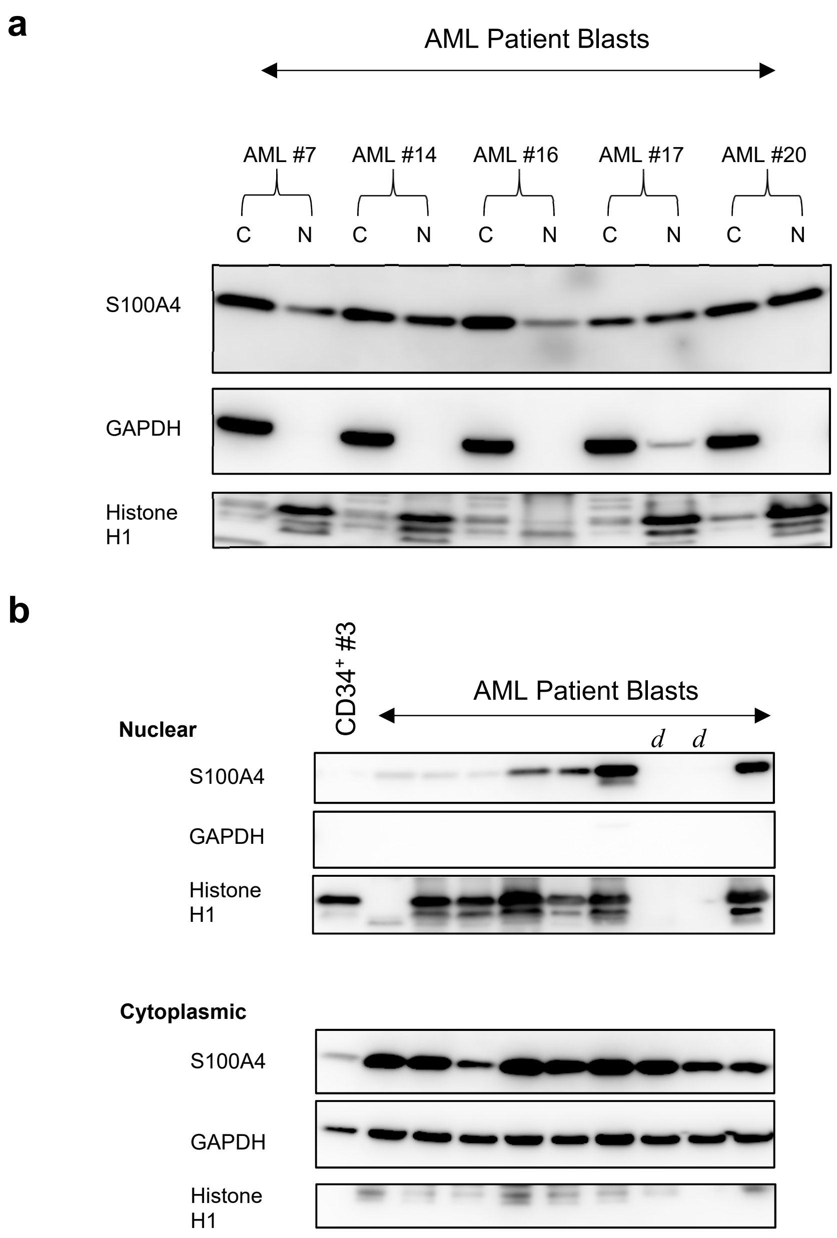

Supplemental Figure S3. S100A4 is over-expressed in the nucleus of AML FAB-M1 patient. Example western blots of PVDF membranes with AML FAB-M1 patient samples analyzed according to sub-cellular fraction. **(a)** To compare S100A4 protein expression in the cytosol and nucleus of AML patient samples (on the same membrane with luminescence captured under identical conditions), a selection of random samples (using samples from MS analysis) was immunoblotted with cytosol and nuclear extracts. **(b)** Further validation of S100A4 protein expression in a random selection of AML patient samples. Cytosolic (C) and nuclear (N) fractions were analysed by GAPDH and Histone to indicate the purity/relative loading of each fraction.

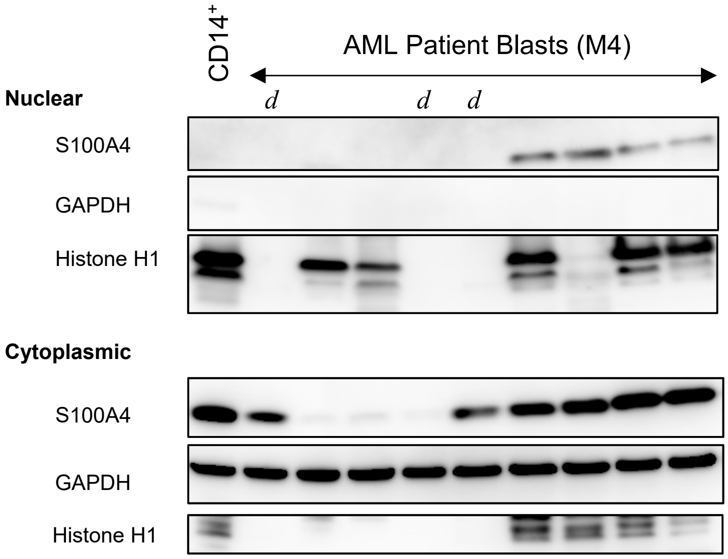

Supplemental Figure S4. S100A4 is over-expressed in the nucleus of a cohort of FABM4-AML patients. Cytosolic and nuclear fractions were analysed by GAPDH and Histone H1 to indicate the purity/relative loading of each fraction. *d* represents degraded nuclear protein based on Histone H1 immunoblot.

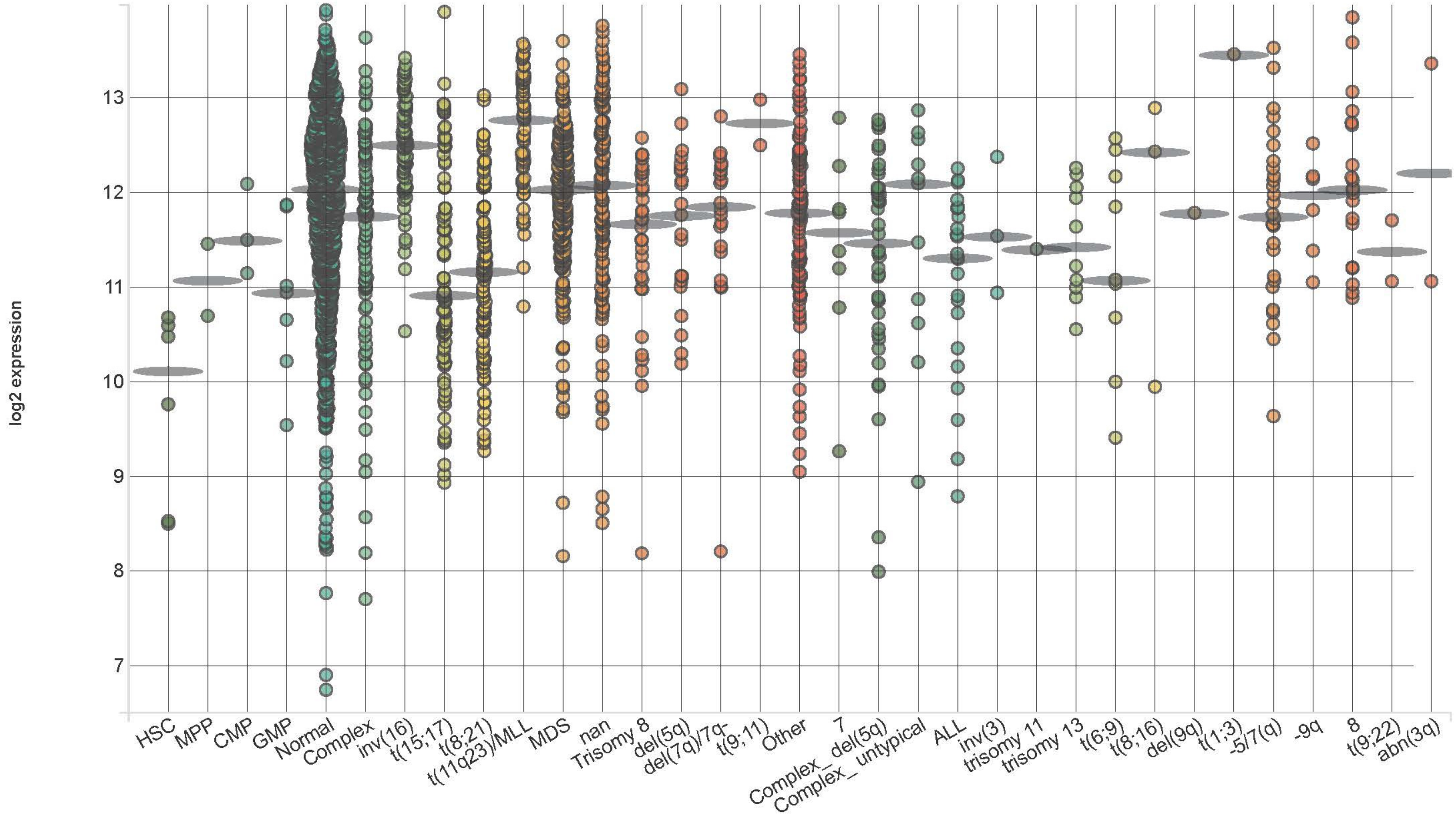

Supplemental Figure S5. Over-expression of *S100A4* mRNA in AML. *S100A4* mRNA expression data analysed using Bloodspot (http://servers.binf.ku.dk/bloodspot/) [1] and published online datasets. Human normal hematopoiesis data derived from GSE42519 (ref. [1]). Data from Human AML blasts are derived from GSE13159 (ref. [2]), GSE15434 (ref. [3]), GSE61804 (ref. [4]), GSE14468 (ref. [5]) and The Cancer Genome Atlas (TCGA) [6]. HSC, Hematopoietic stem cell Lin$^-$ CD34$^+$ CD38$^-$ CD90$^+$ CD45RA$^-$; MPP, Multipotential progenitors Lin$^-$ CD34$^+$ CD38$^-$ CD90$^-$ 45RA$^-$; CMP, Common myeloid progenitors cell Lin$^-$ CD34$^+$ CD38$^+$ CD45RA$^-$ CD123$^+$; GMP, Granulocyte monocyte progenitors Lin$^-$ CD34$^+$ CD38$^+$ CD45RA$^+$ CD123$^+$. Human AML subtype definitions are shown over-page. References are at the end of this document.

Normal	AML with Normal karyotype
Complex	AML with Complex karyotype
inv(16)	AML with inv(16)
t(15;17)	AML with t(15;17)
t(8;21)	AML with t(8;21)
t(11q23)/MLL	AML with t(11q23)/MLL
MDS	MDS
nan	AML with no karyotype information
Trisomy 8	AML with Trisomy 8
del(5q)	AML with del(5q)
del(7q)/7q-	AML with del(7q)/7q-
t(9;11)	AML with t(9;11)
Other	AML with Other abnormalities
7	AML with +7
Complex_ del(5q)	AML with Complex del(5q)
Complex_ untypical	AML with Complex untypical karyotype
ALL	ALL
inv(3)	AML with inv(3)
trisomy 11	AML with trisomy 11
trisomy 13	AML with trisomy 13
t(6;9)	AML with t(6;9)
t(8;16)	AML with t(8;16)
del(9q)	AML with del(9q)
t(1;3)	AML with t(1;3)
-5/7(q)	AML with -5/7(q)
-9q	AML with -9q
8	AML with +8
t(9;22)	AML with t(9;22)
abn(3q)	AML with abn(3q)

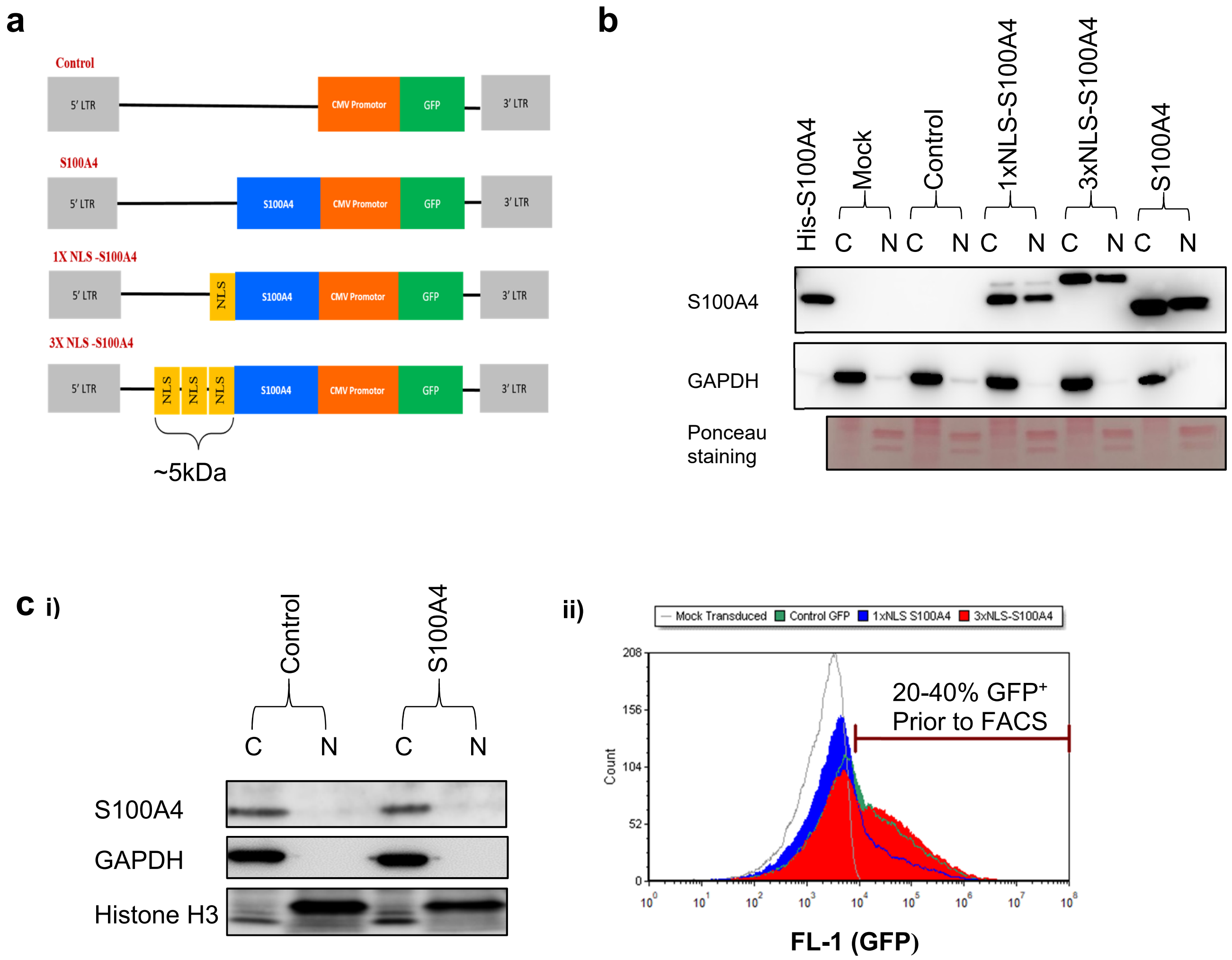

Supplemental Figure S6. Generation and validation of viral constructs of S100A4. (a) Graphical representation of the PINCO retroviral vectors created for this study. S100A4 and nuclear localized signal (NLS)-S100A4 were created by directionally cloning into the *Bam*H1 and *Eco*R1 restriction enzyme sites of the PINCO retroviral vector. NLS; GATCCAAAAAGAAGAGAAAGGTA. Similar lentiviral vectors were created using an alternative pHIV vector (Addgene), though identical outcomes were observed. **(b)** Example western blots showing S100A4 protein expression and subcellular localisation in HEK293T Phoenix^{TM} packaging cells transfected with plasmid cDNA shown in (a). **(c)** (i) Western blot of S100A4 expression in subcellular extracts from cord blood derived CD34^{+} cells transduced with virus encoding GFP alone (control) or S100A4 (lacking NLS sequence) and grown in cytokine-driven culture (IL-3, SCF, G-CSF and GM-CSF in supplemented IMDM) until day 13 and sorted for GFP positivity prior to analysis. Similar results were obtained for NLS based vectors (data not shown). **(c)**(ii) Example flow cytometric histograms showing GFP expression in infected CD34^{+} cells. GFP^{+} was based on background autofluorescence of Mock (GFP^{-}) transduced CD34 cells Cytosolic (C) and nuclear (N) fractions were analyzed by GAPDH and Ponceau to indicate the purity/relative loading of each fraction. FACS; fluorescence activated cell sorting.

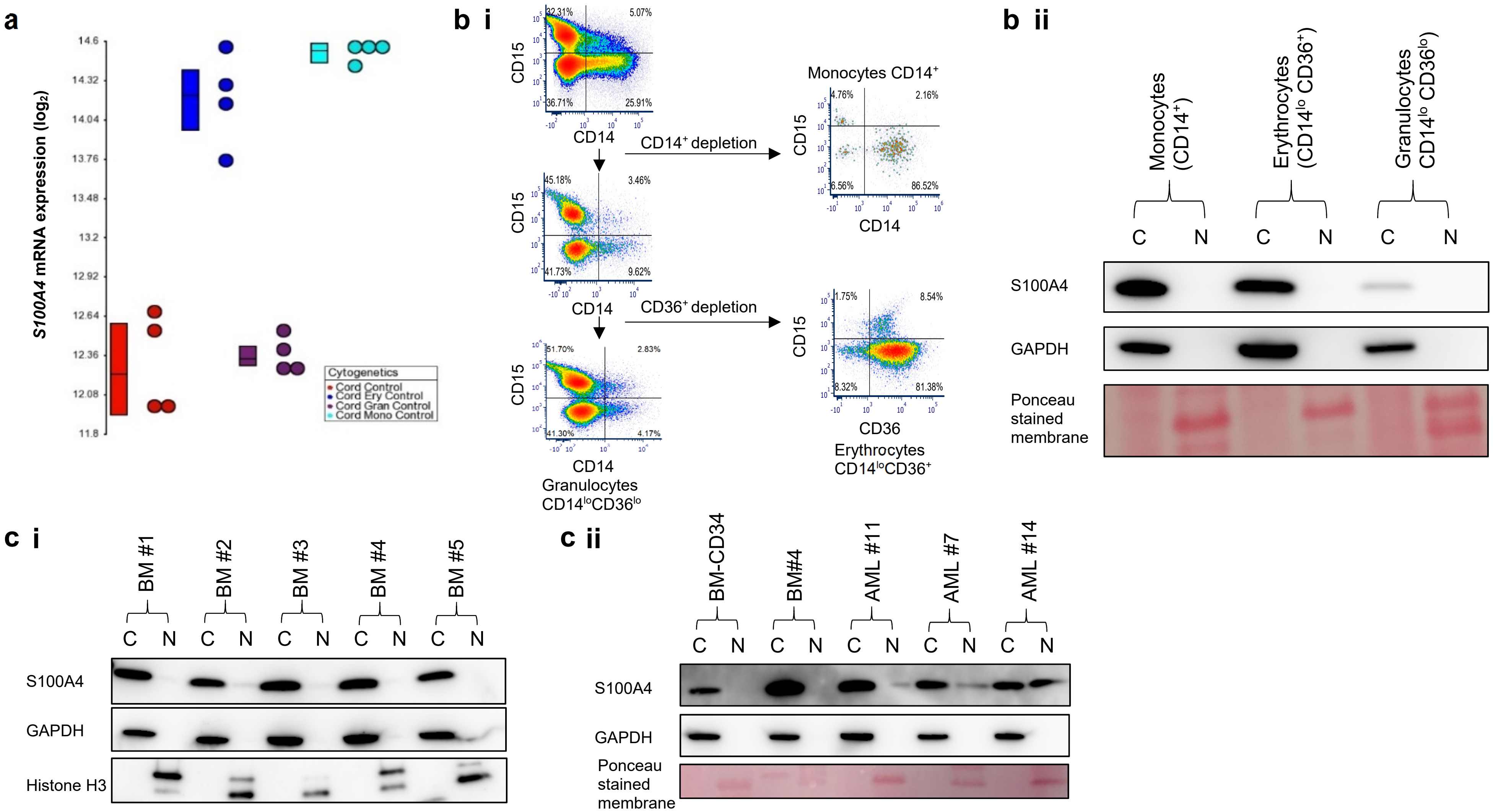

Supplemental Figure S7. S100A4 expression in normal human hemopoietic progenitor cells and differentiated lineages. (a) Microarray data demonstrating the normalized intensity of *S100A4* (log_2) mRNA expression in normal human $CD34^+$ cells (red) and sorted hemopoietic subsets. Erythroid cells, dark blue; granulocytes, brown; monocytes, light blue (n=4). Horizontal lines indicate the average normalized intensity of *S100A4* expression within each sub-type. Data derived using Array Express (E-MEXP-583; from Tonks *et al*, 2007) (ref.[7]). **(b)** (i) Representative flow cytometric density plots of human blood progenitor cells enriched for specific lineages on day 6 of culture using a MiniMACS depletion strategy. Quadrants delimit background fluorescence of control-stained cells. (ii) Western blot showing S100A4 protein expression and subcellular localization in normal human monocytes, erythrocytes and granulocytes isolated through MiniMacs outlined in b(i). **(c)** Western blot showing S100A4 protein expression and subcellular localization in (i) normal human bone marrow (n=5) and (ii) human bone marrow derived $CD34^+$ cells, representative whole human bone marrow and human AML blasts. Cytosolic (C) and nuclear (N) fractions were analysed by GAPDH, Histone and Ponceau staining of the PVDF membrane to indicate the purity/relative loading of each fraction.

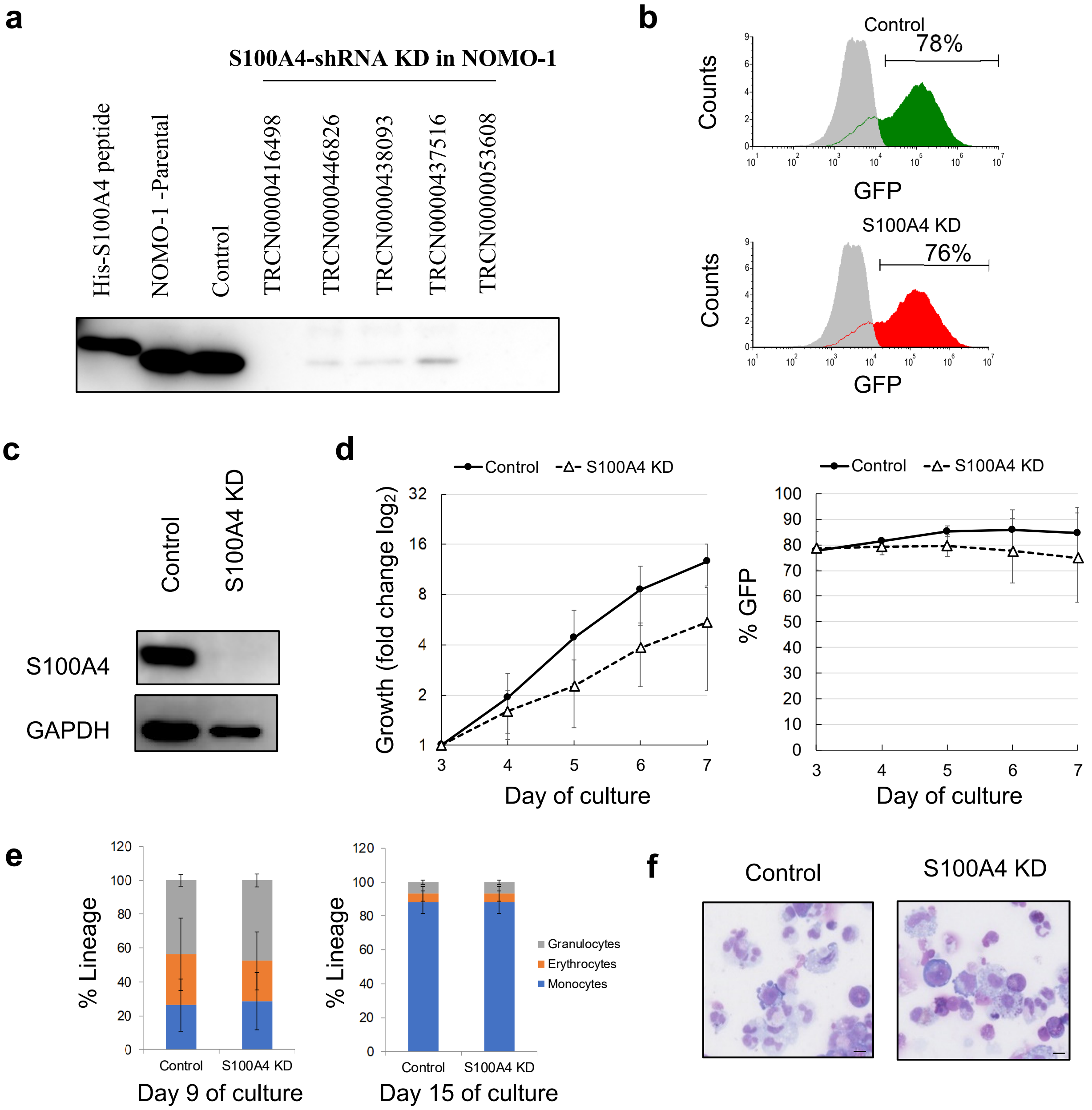

Supplemental Figure S8. S100A4 knock down in CD34$^+$ cells does not affect the development or survival of human myeloid hemopoietic cells. (a) Western blot showing S100A4 knock down (KD) in NOMO-1 cells using a panel of Mission® shRNA vectors (Sigma). Target sequence from TRCN0000416498 was used in subsequent studies due to its S100A4 KD efficiency). Control cells were infected with shRNA targeting a non-mammalian gene. His tagged S100A4 and mock infected NOMO-1 (parental) were used as positive controls. **(b)** Cord blood derived CD34$^+$ cells were transduced with lentivirus (Vector Builder) encoding GFP and shRNA to non-mammalian gene (control) or S100A4 (S100A4 KD) and grown in cytokine-driven culture (IL-3, SCF and G/GM-CSF). Representative flow cytometric histograms showing infection efficiency of CD34$^+$ cells analyzed on day 3 of culture. Mock transduced cultures (gray) delimits background autofluorescence. **(c)** Western blot of S100A4 expression in CD34$^+$ cells grown under myeloid differentiating conditions until day 13 and sorted for GFP$^+$. **(d)** Expansion of shRNA transduced CD34$^+$ cells; fold change (*Left*) and proportion of GFP expression (*Right*) over 7 days. Data indicated mean ± 1SD (n=3). **(e)** Analysis of the differentiation of transduced CD34$^+$ by four-colour cytometric analysis. GFP$^+$ myeloid cells were gated on CD13^{hi},CD36^{lo} (granulocytes), CD13^{hi},CD36^{hi} (monocytes) and CD13^{lo},CD36^{lo} (erythrocytes). Bar chart showing the proportion of cells committed to sub-lineage development during culture. Data from 1 experiment; mean ± 1SD from five intra experimental repeats. **(f)** Cytocentrifuge preparations, stained with Wright-Giemsa following 15 days in culture, showing myeloid morphology in the control (*Left*) and S100A4 KD (*Right*)-transduced cultures. Magnification is ×20 and scale bar represents 10µm.

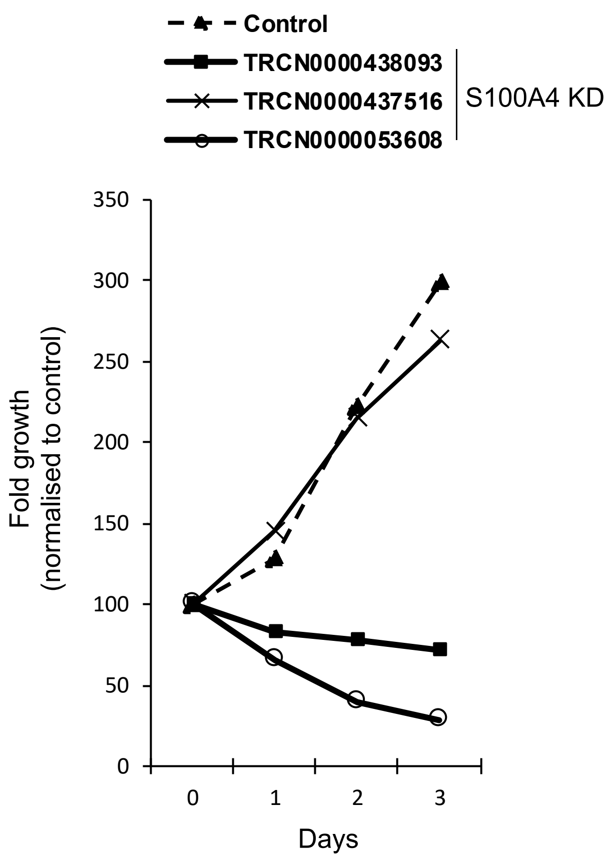

Supplemental Figure S9. S100A4 is required for cell growth in NOMO-1 cells. Data showing growth of NOMO-1 cells using a panel of Mission® shRNA vectors (Sigma). The KD levels of S100A4 protein aare shown in Supplemental Figure S8A. Control cells were infected with shRNA targeting a non-mammalian gene. S100A4 KD was compared to control over 3 days of growth following infection. TCRN numbers identify target shRNA sequences used. Data points indicates mean (n=1).

Reference List

(1) Rapin N, Bagger FO, Jendholm J, Mora-Jensen H, Krogh A, Kohlmann A, *et al*. Comparing cancer vs normal gene expression profiles identifies new disease entities and common transcriptional programs in AML patients. *Blood* 2014 Feb 6;123(6):894-904.

(2) Kohlmann A, Kipps TJ, Rassenti LZ, Downing JR, Shurtleff SA, Mills KI, *et al*. An international standardization programme towards the application of gene expression profiling in routine leukaemia diagnostics: the Microarray Innovations in LEukemia study prephase. *Br J Haematol* 2008 Sep;142(5):802-7.

(3) Klein HU, Ruckert C, Kohlmann A, Bullinger L, Thiede C, Haferlach T, *et al*. Quantitative comparison of microarray experiments with published leukemia related gene expression signatures. *BMC Bioinformatics* 2009 Dec 15;10:422.

(4) Metzelder SK, Michel C, von BM, Rehberger M, Hessmann E, Inselmann S, *et al*. NFATc1 as a therapeutic target in FLT3-ITD-positive AML. *Leukemia* 2015 Jul;29(7):1470-7.

(5) Wouters BJ, Lowenberg B, Erpelinck-Verschueren CA, van Putten WL, Valk PJ, Delwel R. Double CEBPA mutations, but not single CEBPA mutations, define a subgroup of acute myeloid leukemia with a distinctive gene expression profile that is uniquely associated with a favorable outcome. *Blood* 2009 Mar 26;113(13):3088-91.

(6) Ley TJ, Miller C, Ding L, Raphael BJ, Mungall AJ, Robertson A, *et al*. Genomic and epigenomic landscapes of adult de novo acute myeloid leukemia. *N Engl J Med* 2013 May 30;368(22):2059-74.

(7) Tonks A, Pearn L, Tonks AJ, Pearce L, Hoy T, Phillips S, et al. The AML1-ETO fusion gene promotes extensive self-renewal of human primary erythroid cells. *Blood.* 2003;101:624-32.
