# Supplementary material for: Integrated nuclear proteomics and transcriptomics identifies S100A4 as a therapeutic target in acute myeloid leukemia
Source: Leukemia. 2019 Oct 14;34(2):427–40. doi: 10.1038/s41375-019-0596-4 (PMC6995695; doi:10.1038/s41375-019-0596-4)
Supplement: Supplementary file 8 — Table S6 [file 41375_2019_596_MOESM8_ESM.docx]

Supplemental Table S6. *Significantly changing nuclear proteins in AML patient blasts.* The top 10 most significant protein changes are shown based on the *^≠^*product of frequency of observation and magnitude of change. At a minimum, proteins must have significantly changed ± > 2 fold between normal CD34^+^ control and AML in at least 5 of the 15 patients (Frequency) derived from Supplemental Table S2. ^#^ Calculation based solely on patients where a significant change was observed to derive the average fold change of AML *vs* CD34^+^ normal control. Positive fold change values are upregulated in AML *vs* control. Negative values downregulated in AML *vs* control.

| Gene Symbol | Gene Name | Frequency | Fold Change  (AML *vs* CD34^+^)^#^ | Freq*fold change^≠^ | Abnormally expressed in AML^¥^ |
| --- | --- | --- | --- | --- | --- |
| HMGA2 | High mobility group protein HMGI | 14 | -6.0 | 84.7 | Marquis, M., *et al*. High expression of HMGA2 independently predicts poor clinical outcomes in acute myeloid leukemia. ***Blood Cancer J***. 8(8), 68. 19-7-2018  Tan, L., *et al.* Silencing of HMGA2 reverses retardance of cell differentiation in human myeloid leukaemia. ***Br.J.Cancer*** 118(3), 405-415. 6-2-2018. |
| ANXA1 | Annexin A1 | 11 | 6.3 | 69.6 | Kazmierczak, M., *et al.* Esterase D and gamma 1 actin level might predict results of induction therapy in patients with acute myeloid leukemia without and with maturation. ***Med.Oncol.*** 30(4), 725. 2013.  Luczak, M., *et al*. Comparative proteome analysis of acute myeloid leukemia with and without maturation. ***J.Proteomics.*** 75(18), 5734-5748. 22-10-2012. |

| Gene Symbol | Gene Name | Frequency | Fold Change  (AML *vs* CD34^+^)^#^ | Freq*fold change^≠^ | References in AML^¥^ |
| --- | --- | --- | --- | --- | --- |
| PTRF | Polymerase I and transcript release factor | 11 | -5.7 | 63.4 | Lee, S., *et al*. Gene expression profiles in acute myeloid leukemia with common translocations using SAGE. ***Proc.Natl.Acad.Sci.U.S.A*** 103(4), 1030-1035. 2006.  Loke, J., *et al.* C/EBPalpha overrides epigenetic reprogramming by oncogenic transcription factors in acute myeloid leukemia. ***Blood Adv***. 2(3), 271-284. 2018.  de, Boer B *et al*. Prospective Isolation and Characterization of Genetically and Functionally Distinct AML Subclones. ***Cancer Cell*** 34(4), 674-689. 2018. |
| S100A4 | Protein S100-A4 | 11 | 5.5 | 60.5 | Not reported |
| LSP1 | Lymphocyte-specific protein 1 | 13 | -4.2 | 54.8 | Wagner, S., *et al.* A parsimonious 3-gene signature predicts clinical outcomes in an acute myeloid leukemia multicohort study. ***Blood Adv.*** 3(8), 1330-1346. 2019.  Zhao, X.,*et al*. A novel scoring system for acute myeloid leukemia risk assessment based on the expression levels of six genes. ***Int.J.Mol.Med***. 42(3), 1495-1507. 2018 |
| MYEF2 | Myelin expression factor 2 | 10 | -5.4 | 54.1 | de, Boer B *et al*. Prospective Isolation and Characterization of Genetically and Functionally Distinct AML Subclones. ***Cancer Cell*** 34(4), 674-689. 2018. |
| MPO | Myeloperoxidase | 9 | 5.3 | 48.4 | Tominaga-Sato, S., *et al.* Expression of myeloperoxidase and gene mutations in AML patients with normal karyotype: double CEBPA mutations are associated with high percentage of MPO positivity in leukemic blasts. ***Int.J.Hematol***. 94(1), 81-89. 2011.  Itonaga, H., *et al*. Expression of myeloperoxidase in acute myeloid leukemia blasts mirrors the distinct DNA methylation pattern involving the downregulation of DNA methyltransferase DNMT3B. ***Leukemia*** 28(7), 1459-1466. 2014.  de, Boer B *et al*. Prospective Isolation and Characterization of Genetically and Functionally Distinct AML Subclones. ***Cancer Cell*** 34(4), 674-689. 2018. |
| Gene Symbol | **Gene Name** | **Frequency** | **Fold Change**  **(AML vs CD34+)^#^** | **Freq*fold change^≠^** | **References in AML¥** |
| S100A11 | Protein S100-A11 | 9 | 4.5 | 42.8 | Not reported |
| FLNB | Filamin-B | 13 | -3.3 | 42.7 | de, Boer B *et al*. Prospective Isolation and Characterization of Genetically and Functionally Distinct AML Subclones. ***Cancer Cell*** 34(4), 674-689. 2018. |
| CT45A5 | Cancer/testis antigen family 45 member A5 | 5 | 7.21 | 36.0 | de, Boer B *et al*. Prospective Isolation and Characterization of Genetically and Functionally Distinct AML Subclones. ***Cancer Cell*** 34(4), 674-689. 2018. |
